# The TDNNS method for Reissner–Mindlin plates

**DOI:** 10.1007/s00211-017-0883-9

**Published:** 2017-04-27

**Authors:** Astrid S. Pechstein, Joachim Schöberl

**Affiliations:** 1 Institute of Technical Mechanics, Johannes Kepler University Linz, Altenbergerstr. 69, 4040 Linz, Austria; 2 Institute for Analysis and Scientific Computing, Vienna University of Technology, Wiedner Hauptstrasse 8-10, 1040 Wien, Austria

**Keywords:** 65N30, 74K20

## Abstract

A new family of locking-free finite elements for shear deformable Reissner–Mindlin plates is presented. The elements are based on the “tangential-displacement normal-normal-stress” formulation of elasticity. In this formulation, the bending moments are treated as separate unknowns. The degrees of freedom for the plate element are the nodal values of the deflection, tangential components of the rotations and normal–normal components of the bending strain. Contrary to other plate bending elements, no special treatment for the shear term such as reduced integration is necessary. The elements attain an optimal order of convergence.

## Introduction

In this paper we are concerned with finite elements for shear deformable plates based on the Reissner–Mindlin model [34, 42]. A direct discretization of the equations leads to shear locking phenomena as the plate thickness becomes small. In the limit of zero thickness, the Kirchhoff assumption is enforced, where the shear strain vanishes and the deflection gradient equals the rotations. Over the last decades, a vast amount of different elements overcoming shear locking by different kinds of remedies has been proposed. In most standard, conforming finite element methods, the Kirchhoff constraint of vanishing shear stress is alleviated or modified in some way. An alternative are discontinuous Galerkin (DG) methods, mixed, or hybrid methods.

An example for the alleviation of the Kirchhoff constraint is the “assumed shear strain method” introduced by MacNeal [33]. A special operator for the displacement–strain relation relying on local averaging is used, this approach was further developed by Refs. [10, 21, 31]. In a further method referred to as “linked interpolation”, the displacement gradient in the Kirchhoff constraint is augmented by a “kinematic linking operator”. Pioneers in this field were Zienkiewicz et al. [49] and Taylor and Auricchio [47]. Auricchio and Lovadina [9] provide an analysis of general linked-interpolation elements.

Other methods employ additional unknowns for the shear stress quantity, which allows to pose the Kirchhoff condition of vanishing shear in weak sense. Examples are the Falk–Tu element [25] or the MITC element [16]. In implementations, the further unknown can be eliminated element-wise, which leads to a projection of the shear stresses in the penalty term. This projection is referred to as “reduction” in [16]. In some cases, it may be achieved by reduced integration of the shear term. This approach is analyzed minutely for the one-dimensional case of a thick beam in [2]. Reduced integration is also used without the background of an additional shear stress unknown [26, 48], where care has to be taken to avoid spurious modes.

For MITC elements, error analysis has been provided; see e.g. the works by Brezzi et al. [17], where additionally a postprocessing step for the deflections is proposed, or by Stenberg and Suri [46] for an *hp* error analysis.

Nonconforming elements have been constructed by Arnold and Falk [6], where Crouzeix–Raviart elements are used for the deflection. More recently, Brezzi and Marini [18] developed a nonconforming element in the framework of discontinuous Galerkin methods. In both works, the shear strain is projected into a lower-order finite element space to alleviate the Kirchhoff constraint. Other DG approaches allow for a direct enforcement of the Kirchhoff constraint, as the rotation space can be chosen such that it contains the deflection gradient. Deflections, rotations and the shear are approximated using different continuity assumptions in [5]. In [14, 27] DG methods for deflection and rotation without reduced integration techniques are presented. We also mention [19] for a discontinuous Petrov–Galerkin method, where optimal test functions of higher polynomial order are chosen to suit the trial functions. A quadrilateral hybrid finite element method was introduced in [20], where shear stress and bending moment are discontinuous and the corresponding finite element basis is constructed to satisfy a local equilibrium condition.

Hughes and Franca [30] added an additional stabilization term to the variational equations. Also, Chapelle and Stenberg [22] augmented the equations by a stabilization term, which then allows for an analysis ensuring an optimal order of convergence. An entirely different approach by Pontaza and Reddy [41] is to use a least squares method instead of the standard Galerkin equations.

Mixed method with weak symmetry for the tensor of bending moments are proposed in [11, 12]. In both works, the bending moments are approximated in the normal-continuous space ${\underline{\mathbf {H}}}(\mathbf {{div}})$. Since symmetric ${\underline{\mathbf {H}}}(\mathbf {{div}})$-conforming elements are hard to construct and of high polynomial order [8], imposing symmetry weakly using a Lagrange multiplier has often been proposed in the context of continuum mechanics, see e.g. [4, 7, 44, 45]. The continuum mechanics formulation used in the current paper overcomes this problem.

The plate elements proposed in the current paper are based on the “tangential-displacement normal-normal-stress” (TDNNS) formulation of elasticity introduced by the authors in [38]. This leads to a formulation containing deflection, rotations and bending moments as separate unknowns. While the deflection is sought in the standard Sobolev space $H^1$, the rotation is assumed to be in the less regular space ${\mathbf {H}({\text {curl}})}$. Additionally the TDNNS stress space ${\underline{\mathbf {H}}({\text {div}}\,\mathbf {{div}})}$ is chosen for the bending moments. Accordingly, we use standard continuous finite elements for the deflection, tangential continuous Nédélec elements for the rotations, and normal–normal continuous tensor valued elements from [38] for the bending moments. The main benefit of this choice is that the gradient of the deflection space is a subset of the rotation space both for the infinite dimensional and for the finite element problem, and the Kirchhoff constraint of vanishing shear strain does not lead to locking. Thus, the proposed formulation seems to be a very natural alternative to the established ones based on $H^1$ continuity and continuous finite elements. In [39] the authors have shown that three-dimensional anisotropic TDNNS elements are suitable for the discretization of slim domains. The proposed Reissner–Mindlin plate elements show an optimal order of convergence, which is confirmed in our numerical results.

The proposed elements are closely related to the Hellan–Herrmann–Johnson [28, 29, 32] element for the bending problem of a Kirchhoff plate. Also in the HHJ formulation, the normal–normal component of the bending moment is continuous across interfaces. As the Hellan–Herrmann–Johnson element is restricted to the biharmonic problem, the rotations are not treated independently as done in the current work. On the other hand, for vanishing thickness one can eliminate the rotations in the current formulation, and arrives at the HHJ plate formulation. Thus, the HHJ formulation may be seen as the limiting case of the proposed method. In [24], Lagrangian multipliers for the normal–normal component of the bending moment are introduced. Postprocessing then leads to a faster convergence of the deflection gradient.

This work is organized as follows: in Sect. 2, the TDNNS method is shortly introduced and applied to the Reissner–Mindlin problem. An analysis of the infinite dimensional problem using the TDNNS spaces for positive as well as vanishing thickness is provided in Sect. 3. Finite elements are introduced in Sect. 4, and a-priori error estimates are provided. We mention hybridization of the bending moments by Lagrangian multipliers resembling the normal component of the rotation, which results after static condensation in a symmetric positive system matrix. Finally, Sect. 5 contains numerical examples verifying the claimed convergence orders.

## Problem formulation

### Notation

In the following, all vectors are denoted by boldface letters, tensors are boldface and underlined. Let $\varOmega \subset \mathbb R^2$ be a bounded, connected, polygonal Lipschitz domain. Its unit outward normal $\mathbf {n}$ is defined almost everywhere on the boundary $\partial \varOmega$. The unit tangential vector in counter-clockwise direction ${\varvec{\tau }}$ is given as the rotation of $\mathbf {n}$,

1 $$\begin{aligned} {\varvec{\tau }}= \mathbf {n}^\bot = \left( \begin{array}{c}-n_y\\ n_x\end{array} \right) . \end{aligned}$$

For a vector field $\mathbf {v}$ on $\varOmega$, normal and tangential component on the boundary are denoted by

2 $$\begin{aligned} v_n := \mathbf {v}\cdot \mathbf {n},\qquad \mathbf {v}_{\varvec{\tau }}= \mathbf {v}- v_n \mathbf {n}. \end{aligned}$$

For a tensor field of second order $\underline{\varvec{\sigma }}$, its normal component is given by $\varvec{\sigma }_\mathbf {n}:= \underline{\varvec{\sigma }}\mathbf {n}$. The normal component $\varvec{\sigma }_\mathbf {n}$ can further be split into a (scalar-valued) normal–normal component $\sigma _{nn}$ and a (vector-valued) normal–tangential component $\varvec{\sigma }_{\mathbf {n}{\varvec{\tau }}}$,

3 $$\begin{aligned} \sigma _{nn} = \varvec{\sigma }_\mathbf {n}\cdot \mathbf {n}, \qquad \varvec{\sigma }_{\mathbf {n}{\varvec{\tau }}} = \varvec{\sigma }_\mathbf {n}- \sigma _{nn} \mathbf {n}. \end{aligned}$$

Rotation and divergence of a two-dimensional vector field shall be denoted by ${\text {curl}}$, ${\text {div}}$, respectively. The operator $\mathbf {{div}}$ is the row-wise divergence operator, mapping tensor to vector fields.

For a general Hilbert space *V*, its inner product and norm are denoted by $(\cdot ,\cdot )_V$ and $\Vert \cdot \Vert _V$, respectively. The duality product between *V* and its dual $V^*$ is denoted by angles,

4 $$\begin{aligned} \langle f, g \rangle = f(g) \qquad \forall f \in V^*, g \in V. \end{aligned}$$

Let $L^2(\varOmega )$ denote the usual Lebesgue space. Moreover, let $H^1(\varOmega )$ be the usual Sobolev space of weakly differentiable functions, and let $H^1_0(\varOmega )$ be the space of $H^1$ functions satisfying zero boundary conditions. We also use the Sobolev spaces $H^s(\varOmega )$ for integer *s*. Moreover, ${\mathbf {H}({\text {curl}},\varOmega )}$ and ${\mathbf {H}_0({\text {curl}},\varOmega )}$ shall be the spaces of vector-valued functions with weak rotation, the latter satisfying zero boundary conditions for the tangential component of the vector fields, see [35].

The dual space of $H^1_0(\varOmega )$ shall be denoted by $H^{-1}(\varOmega )$. It is well established [13, Equation (10.4.52)] that the dual space of ${\mathbf {H}_0({\text {curl}},\varOmega )}$ is ${\mathbf {H}^{-1}({\text {div}},\varOmega )}$ being the space of $\mathbf {H}^{-1}$ vector fields with distributional divergence in $H^{-1}$,

5 $$\begin{aligned} {\mathbf {H}^{-1}({\text {div}},\varOmega )}= \{ \mathbf {f}\in \mathbf {H}^{-1}(\varOmega ): {\text {div}}\mathbf {f}\in H^{-1}(\varOmega )\} = ({\mathbf {H}_0({\text {curl}},\varOmega )})^* . \end{aligned}$$

The distributional divergence operator is defined by the relationship

6 $$\begin{aligned} \langle {\text {div}}\,\mathbf {f}, w\rangle = -\langle \mathbf {f}, \nabla w\rangle \qquad \forall w \in C^\infty _0(\varOmega ). \end{aligned}$$

Using this definition, a natural norm of ${\mathbf {H}^{-1}({\text {div}},\varOmega )}$ is

7 $$\begin{aligned} \Vert \mathbf {f}\Vert _{{\mathbf {H}^{-1}({\text {div}},\varOmega )}}^2= & {} \Vert \mathbf {f}\Vert _{{\mathbf {H}}^{-1}(\varOmega )}^2 + \Vert {\text {div}}\mathbf {f}\Vert _{H^{-1}(\varOmega )}^2 \end{aligned}$$

8 $$\begin{aligned}= & {} \sup _{\mathbf {v}\in {\mathbf {H}}^1_0(\varOmega )} \frac{\langle \mathbf {f}, \mathbf {v}\rangle ^2}{\Vert \nabla \mathbf {v}\Vert _{{\underline{\mathbf {L}}}^2(\varOmega )}^2} + \sup _{w \in H^1_0(\varOmega )} \frac{\langle \mathbf {f}, \nabla w\rangle ^2}{\Vert \nabla w\Vert _{{\mathbf {L}}^2(\varOmega )}^2}. \end{aligned}$$

In all further occurrences of Sobolev spaces on $\varOmega$, the domain can also be omitted. We thus write $H^1$ for $H^1(\varOmega )$ or ${\mathbf {H}({\text {curl}})}$ for ${\mathbf {H}({\text {curl}},\varOmega )}$. When defined on domains other than $\varOmega$, the domain must be indicated as above.

### The TDNNS method

In [38, 39] we introduced the tangential-displacement normal-normal-stress (TDNNS) method for elasticity, and refined the analysis in [40]. In the current section, we briefly cover the main idea of the TDNNS method for elasticity problems in the two-dimensional continuum, as the proposed Reissner–Mindlin elements will be based on this method.

Let $\varOmega \subset \mathbb R^2$ be a bounded, connected, polygonal domain with Lipschitz boundary $\partial \varOmega$. The displacement vector $\mathbf {u}= (u_1,u_2)$ and symmetric stress tensor $\underline{\varvec{\sigma }}\in \mathbb R^{2\times 2}_{sym}$ are connected via Hooke’s law (9) and the equilibrium equation (10)

9 $$\begin{aligned} \underline{\mathbf {A}}\underline{\varvec{\sigma }}&= \underline{\varvec{\varepsilon }}(\mathbf {u})&\text{ in } \varOmega , \end{aligned}$$

10 $$\begin{aligned} \mathbf {{div}}\underline{\varvec{\sigma }}&= -\mathbf {f}&\text{ in } \varOmega . \end{aligned}$$

Here, we use the linearized strain tensor $\underline{\varvec{\varepsilon }}(\mathbf {u}) = \frac{1}{2} (\nabla \mathbf {u}+ (\nabla \mathbf {u})^T)$. Stress and strain are connected via the compliance tensor $\underline{\mathbf {A}}$, which is the inverse of the elasticity tensor $\underline{\mathbf {C}}$ depending on Young’s modulus *E* and the Poisson ratio $\nu$ in the well known way. For simplicity, we assume that homogeneous displacement boundary conditions are posed on $\partial \varOmega$,

11 $$\begin{aligned} \mathbf {u}= 0 \qquad \text{ on } \partial \varOmega . \end{aligned}$$

Most standard methods for the elasticity problem rely on a primal formulation, which is obtained eliminating the stress tensor $\underline{\varvec{\sigma }}$ from Eqs. (9) and (10). Then one searches for the displacement $\mathbf {u}$ in $\mathbf {H}^1_0$ such that

12 $$\begin{aligned} \int _\varOmega \underline{\mathbf {C}}\underline{\varvec{\varepsilon }}(\mathbf {u}):\underline{\varvec{\varepsilon }}(\mathbf {v})\,dx = \int _\varOmega \mathbf {f}\cdot \mathbf {v}\,dx \qquad \forall \mathbf {v}\in \mathbf {H}^1_0. \end{aligned}$$

In a conforming finite element method, the displacement vector $\mathbf {u}$ is approximated by a continuous finite element function.

On the other hand, the dual Hellinger–Reissner formulation is obtained directly from system (9), (10) when multiplying with test functions and using integration by parts,

13 $$\begin{aligned} \int _\varOmega \underline{\mathbf {A}}\underline{\varvec{\sigma }}: \underline{\varvec{\tau }}\, dx + \int _\varOmega \mathbf {{div}}\underline{\varvec{\tau }}\cdot \mathbf {u}\, dx&= 0&\forall \underline{\varvec{\tau }}\in \underline{\mathbf {H}}_{sym}(\mathbf {{div}}), \end{aligned}$$

14 $$\begin{aligned} \int _\varOmega \mathbf {{div}}\underline{\varvec{\sigma }}\cdot \mathbf {v}\, dx&= \int _\varOmega \mathbf {f}\cdot \mathbf {v}\, dx&\forall \mathbf {v}\in \mathbf {L}^2. \end{aligned}$$

Here, the solution spaces are $\underline{\mathbf {H}}_{sym}(\mathbf {{div}})$ for the stress and $\mathbf {L}^2$ for the displacement. In a conforming finite element method, the displacement elements can be totally discontinuous, while the stress elements need to be tensor-valued symmetric and normal-continuous. These requests lead to computationally expensive finite elements of at least 24 degrees of freedom in two dimensions (see [8]) and 162 degrees of freedom in three dimensions (see [1, 3]).

The TDNNS formulation is “in between” those two concepts, where the displacements are not assumed totally continuous or discontinuous, but where the tangential component is assumed to be continuous across element borders. In a mathematical setting, the displacement space is chosen as

15 $$\begin{aligned} {\mathbf {H}_0({\text {curl}})}= \{ \mathbf {v}\in {\mathbf {H}({\text {curl}})}: \mathbf {v}_{\varvec{\tau }}= 0 \text{ on } \partial \varOmega \}. \end{aligned}$$

In [38, 40] we have shown that the corresponding stress space is the space of symmetric ${\underline{\mathbf {L}}}^2$ tensors with weak divergence in the dual space of ${\mathbf {H}_0({\text {curl}})}$. Due to (5), the stress space is given by

16 $$\begin{aligned} {\underline{\mathbf {H}}({\text {div}}\,\mathbf {{div}})}= \left\{ \underline{\varvec{\tau }}\in {\underline{\mathbf {L}}}^2_{sym}: {\text {div}}\mathbf {{div}}\underline{\varvec{\tau }}\in H^{-1}\right\} . \end{aligned}$$

The duality product $\langle \mathbf {{div}}\underline{\varvec{\tau }}, \mathbf {v}\rangle$, where the divergence of a stress tensor is applied to a displacement field, plays an important role in the TDNNS method. In [40] we elaborated on the meaning of the duality product $\langle \mathbf {{div}}\underline{\varvec{\tau }}, \mathbf {v}\rangle$ for $\underline{\varvec{\tau }}\in {\underline{\mathbf {H}}({\text {div}}\,\mathbf {{div}})}$ and $\mathbf {v}\in {\mathbf {H}_0({\text {curl}})}$. We state that, for smooth $\underline{\varvec{\tau }}\in {\underline{\mathbf {H}}({\text {div}}\,\mathbf {{div}})}$ and $\mathbf {v}\in {\mathbf {H}_0({\text {curl}})}$, i.e. for $\mathbf {v}$ smooth with vanishing tangential component $\mathbf {v}_{\varvec{\tau }}= 0$ on $\partial \varOmega$, the duality product can be evaluated by

17 $$\begin{aligned} \langle \mathbf {{div}}\underline{\varvec{\tau }}, \mathbf {v}\rangle = -\int _\varOmega \underline{\varvec{\tau }}: \underline{\varvec{\varepsilon }}(\mathbf {v})\,dx + \int _{\partial \varOmega } \tau _{nn} v_n\, ds. \end{aligned}$$

A natural norm of the stress space ${\underline{\mathbf {H}}({\text {div}}\,\mathbf {{div}})}$ uses this duality product and is given by, see [40]

18 $$\begin{aligned} \Vert \underline{\varvec{\tau }}\Vert _{{\underline{\mathbf {H}}({\text {div}}\,\mathbf {{div}})}}^2 = \Vert \underline{\varvec{\tau }}\Vert _{{\underline{\mathbf {L}}}^2}^2 + \Vert {\text {div}}\mathbf {{div}}\underline{\varvec{\tau }}\Vert _{H^{-1}}^2 = \Vert \underline{\varvec{\tau }}\Vert _{{\underline{\mathbf {L}}}^2}^2 + \sup _{w \in H^1_0 \cap H^2} \frac{\langle \mathbf {{div}}\underline{\varvec{\tau }}, \nabla w\rangle ^2}{\Vert \nabla w\Vert _{{\mathbf {L}}^2}^2}. \end{aligned}$$

It is well known that finite elements for ${\mathbf {H}({\text {curl}})}$ have to be tangential continuous, such as Nédélec elements introduced in [36, 37]. In [38] it was shown that finite elements for the stress space ${\underline{\mathbf {H}}({\text {div}}\,\mathbf {{div}})}$ are normal–normal continuous, meaning that the normal component $\sigma _{nn}$ of the normal stress vector $\varvec{\sigma }_\mathbf {n}$ is continuous across element borders.

One obtains a variational problem of a form similar to the dual problem (13)–(14),

19 $$\begin{aligned} \int _\varOmega \underline{\mathbf {A}}\underline{\varvec{\sigma }}: \underline{\varvec{\tau }}\,dx + \langle \mathbf {{div}}\underline{\varvec{\tau }}, \mathbf {u}\rangle&= 0&\forall \underline{\varvec{\tau }}\in {\underline{\mathbf {H}}({\text {div}}\,\mathbf {{div}})}, \end{aligned}$$

20 $$\begin{aligned} \langle \mathbf {{div}}\underline{\varvec{\sigma }}, \mathbf {v}\rangle&= \int _\varOmega \mathbf {f}\cdot \mathbf {v}\,dx&\forall \mathbf {v}\in {\mathbf {H}_0({\text {curl}})}. \end{aligned}$$

In a finite element method, it is necessary to evaluate duality products of the form $\langle \mathbf {{div}}\underline{\varvec{\tau }}, \mathbf {v}\rangle$ for piecewise smooth functions with $\tau _{nn}$ and $\mathbf {v}_{\varvec{\tau }}$ continuous on a finite element mesh $\mathcal T = \{T\}$. In this case, the definition from (17) can be extended to

21 $$\begin{aligned} \langle \mathbf {{div}}\underline{\varvec{\tau }}, \mathbf {v}\rangle= & {} \sum _{T \in \mathcal T} \Big ( \int _T \mathbf {{div}}\underline{\varvec{\tau }}\cdot \mathbf {v}\,dx - \int _{\partial T} {\varvec{\tau }}_{\mathbf {n}{\varvec{\tau }}} \cdot \mathbf {v}_{\varvec{\tau }}\,ds \Big ) \end{aligned}$$

22 $$\begin{aligned}= & {} -\sum _{T \in \mathcal T} \Big ( \int _T \underline{\varvec{\tau }}: \underline{\varvec{\varepsilon }}(\mathbf {v})\,dx - \int _{\partial T} \tau _{nn} \cdot v_n\,ds \Big ), \end{aligned}$$

It was shown (see [38, 40]) that the infinite dimensional problem (19), (20) is well posed. Moreover, a stable family of mixed finite elements was constructed, using Nédélec’s elements for the displacement space and a new family of tensor-valued symmetric normal–normal continuous elements for the stress space. The two-dimensional mixed finite elements shall be used in the Reissner–Mindlin elements proposed in this work.

### Reissner–Mindlin model

Let again $\varOmega \subset \mathbb R^2$ be a bounded, connected domain with Lipschitz boundary $\partial \varOmega$. We consider a plate of thickness *t* corresponding to the three-dimensional domain $\varOmega \times (-t/2,t/2)$. In the Reissner–Mindlin model, the displacement vector $\mathbf {u}$ is assumed to take the form

23 $$\begin{aligned} \mathbf {u}= \left( \begin{array}{c}-z\theta _1\\ -z\theta _2\\ w\end{array} \right) , \end{aligned}$$

where $\varvec{\theta }= (\theta _1, \theta _2)$ are rotations and *w* is the deflection in vertical *z* direction. Both the rotations $\varvec{\theta }$ and the deflection *w* are assumed to depend on the in-plane coordinates $(x_1, x_2)$ only.

Assuming a vertical volume load $\mathbf {f}= (0,0,t^2 g)^T \in {\mathbf {L}}^2$ to be given, the Reissner–Mindlin problem for a clamped plate is to find the deflection *w* and rotations $\varvec{\theta }$ such that

24 $$\begin{aligned} -\mathbf {{div}}( \underline{\mathbf {C}}_b\underline{\varvec{\varepsilon }}(\varvec{\theta })) - \mu t^{-2}(\nabla w - \varvec{\theta })&= 0&\text{ in } \varOmega , \end{aligned}$$

25 $$\begin{aligned} -\mu t^{-2}{\text {div}}(\nabla w - \varvec{\theta })&= g&\text{ in } \varOmega , \end{aligned}$$

26 $$\begin{aligned} \varvec{\theta }&= 0&\text{ on } \partial \varOmega , \end{aligned}$$

27 $$\begin{aligned} w&= 0&\text{ on } \partial \varOmega . \end{aligned}$$

Here $\underline{\mathbf {C}}_b$ is the tensor of bending moduli and $\mu$ is the shear modulus with shear correction factor $k_s$, which depend on Young’s modulus *E* and Poisson’s ratio $\nu$ via

28 $$\begin{aligned} \underline{\mathbf {C}}_b= \frac{E}{12(1-\nu ^2)}\left( \begin{array}{ccc} 1 &{}\quad \nu &{}\quad 0 \\ \nu &{}\quad 1 &{}\quad 0 \\ 0 &{}\quad 0 &{}\quad \frac{1-\nu }{2} \end{array} \right) ,\qquad \mu = \frac{k_s E}{2(1+\nu )}. \end{aligned}$$

Additionally, we provide the compliance tensor $\underline{\mathbf {A}}_b= \underline{\mathbf {C}}_b^{-1}$

29 $$\begin{aligned} \underline{\mathbf {A}}_b= \underline{\mathbf {C}}_b^{-1} = \frac{12}{E}\left( \begin{array}{ccc} 1 &{}\quad -\nu &{}\quad 0 \\ -\nu &{}\quad 1 &{}\quad 0 \\ 0 &{}\quad 0 &{}\quad 2(1+\nu ) \end{array} \right) . \end{aligned}$$

Of course, other boundary conditions such as simply supported or free boundaries, or boundary tractions and moments, may be prescribed. Although the analysis of the proposed finite element formulation is done for the clamped case for sake of simplicity, we shall comment shortly on the implementation of other boundary conditions in the end of the current section. We will see that all common types of boundary conditions can be treated in a very natural way.

As in the continuum problem in Sect. 2.2, a primal, displacement-based variational formulation of the Reissner–Mindlin problem (24)–(27) can be obtained directly. Both the rotations $\varvec{\theta }$ and the deflection *w* are assumed weakly differentiable, with $\varvec{\theta }\in \mathbf {\Theta }_{prim} = \mathbf {H}^1_0$ and $w \in W_{prim} = H^1_0$. The primal variational formulation of the Reissner–Mindlin problem is to find $\varvec{\theta }\in \mathbf {\Theta }_{prim}$ and $w \in W_{prim}$ such that for all test functions $\varvec{\eta }\in \mathbf {\Theta }_{prim}$ and $v \in W_{prim}$

30 $$\begin{aligned} \int _\varOmega \underline{\mathbf {C}}_b\underline{\varvec{\varepsilon }}(\varvec{\theta }):\underline{\varvec{\varepsilon }}(\varvec{\eta })\,dx + \mu t^{-2}\int _\varOmega (\nabla w - \varvec{\theta })\cdot (\nabla v - \varvec{\eta })\,dx= & {} \int _\varOmega gv\,dx . \end{aligned}$$

A straightforward finite element discretization of this primal problem choosing continuous finite element spaces $\mathbf {\Theta }_{prim,h} \subset \mathbf {\Theta }_{prim}$ and $W_{prim,h} \subset W_{prim}$ leads to shear locking as the thickness *t* tends to zero. In the limit case of a Kirchhoff plate with $t=0$, the condition of vanishing shear strain

31 $$\begin{aligned} \nabla w - \varvec{\theta }= 0 \end{aligned}$$

has to be satisfied. For conventional finite element discretizations one observes that $\nabla W_{prim,h} \not \subset \mathbf {\Theta }_{prim,h}$, thus the Kirchhoff constraint (31) cannot be satisfied by the discrete solution, the formulation locks. Different methods have been proposed to reduce this phenomenon by alleviating the Kirchhoff constraint (31), see Sect. 1. In this work, the rotation space will be chosen such that both $\nabla W\subset \mathbf {\Theta }$ and $\nabla W_h \subset \mathbf {\Theta }_h$. This ensures stability and an optimal order of convergence of the method.

The main idea of the current work is to use the TDNNS method presented in Sect. 2.2 for the discretization of rotations $\varvec{\theta }$. To this end, additional unknowns $\underline{\mathbf {m}}= \underline{\mathbf {C}}_b\underline{\varvec{\varepsilon }}(\varvec{\theta })$ for the tensor of bending moments are introduced. This leads to the following system of partial differential equations

32 $$\begin{aligned} \underline{\mathbf {A}}_b\underline{\mathbf {m}}- \underline{\varvec{\varepsilon }}(\varvec{\theta })&= 0&\text{ in } \varOmega , \end{aligned}$$

33 $$\begin{aligned} -\mathbf {{div}}( \underline{\mathbf {m}}) - \mu t^{-2}(\nabla w - \varvec{\theta })&= 0&\text{ in } \varOmega , \end{aligned}$$

34 $$\begin{aligned} -\mu t^{-2}{\text {div}}(\nabla w - \varvec{\theta })&= g&\text{ in } \varOmega , \end{aligned}$$

Now, we obtain a variational formulation for finding $\varvec{\theta }\in \mathbf {\Theta }= {\mathbf {H}_0({\text {curl}})}$, $\underline{\mathbf {m}}\in \underline{\mathbf {M}}= {\underline{\mathbf {H}}({\text {div}}\,\mathbf {{div}})}$ and $w \in W= H^1_0$ in the same manner as in Sect. 2.2,

35 $$\begin{aligned} \int _\varOmega \underline{\mathbf {A}}_b\underline{\mathbf {m}}: \underline{\varvec{\tau }}\,dx + \langle \mathbf {{div}}\underline{\varvec{\tau }}, \varvec{\theta }\rangle&= 0&\forall \underline{\varvec{\tau }}\in \underline{\mathbf {M}}, \end{aligned}$$

36 $$\begin{aligned} \langle \mathbf {{div}}\underline{\mathbf {m}}, \varvec{\eta }\rangle - \mu t^{-2} \int _\varOmega (\nabla w - \varvec{\theta })\cdot (\nabla v - \varvec{\eta })\,dx&= -\int _\varOmega gv\,dx&\forall \varvec{\eta }\in \mathbf {\Theta }, v \in W. \end{aligned}$$

We shortly comment on the changes in the variational formulation (35)–(36) which are necessary for the incorporation of different types of boundary conditions. Essential boundary conditions, which have to be enforced by the finite element space, are the deflection *w*, the tangential component of the rotation $\varvec{\theta }_{\varvec{\tau }}$ and the normal component of the bending moment $m_{nn}$. Note that these expressions are also degrees of freedom of the corresponding finite element spaces. The corresponding natural conditions, in the same order, are the shear $\mu t^{-2}(\partial _n w - \theta _n)$, the tangential component of the bending moment $\mathbf {m}_{nt}$, and the normal component of the rotation $\theta _n$. Natural homogeneous conditions are satisfied whenever the corresponding essential condition is dropped, inhomogeneous conditions result in additional surface integrals on the right hand side, see Table 1.

**Table 1:** Different types of essential and corresponding natural boundary conditions and the incorporation of the latter into the right hand side of (35)–(36)

Essential bc	Natural bc	Surface term
*w*	$\mu t^{-2} (\partial _n w - \theta _n) = g_0$	$\int _\varGamma g_0\, v\, ds$
$\varvec{\theta }_{\varvec{\tau }}$	$\mathbf {m}_{\mathbf {n}{\varvec{\tau }}} = \mathbf {g}_1$	$\int _\varGamma \mathbf {g}_1 \cdot \varvec{\eta }_{\varvec{\tau }}\, ds$
$m_{nn}$	$\theta _n = g_2$	$\int _\varGamma g_2\, \tau _{nn}\, ds$

The analysis of system (35), (36) is subject of Sect. 3, while a finite element method is constructed and analyzed in Sect. 4.

## Analysis of the TDNNS Reissner–Mindlin formulation

In the current section we show existence and uniqueness of the solution to the Reissner–Mindlin problem in the TDNNS setting. We show stability for decreasing thickness $t \rightarrow 0^+$ as well as the limit case $t=0$.

To this end, a further unknown $\varvec{\gamma }= -\mu t^{-2} (\nabla w - \varvec{\theta })$ related to the shear stresses is introduced, see e.g. [13, Chapter 10.4]. For positive thickness *t*, we choose the corresponding space $\mathbf {\Gamma }= {\mathbf {L}^2(\varOmega )}$. Problem (35), (36) transforms to

37 $$\begin{aligned} \int _\varOmega \underline{\mathbf {A}}_b\underline{\mathbf {m}}: \underline{\varvec{\tau }}\,dx + \langle \mathbf {{div}}\underline{\varvec{\tau }}, \varvec{\theta }\rangle&= 0&\forall \underline{\varvec{\tau }}\in \underline{\mathbf {M}}, \end{aligned}$$

38 $$\begin{aligned} \langle \mathbf {{div}}\underline{\mathbf {m}}, \varvec{\eta }\rangle + \int _\varOmega \varvec{\gamma }\cdot (\nabla v - \varvec{\eta })\,dx&= -\int _\varOmega gv\,dx&\forall \varvec{\eta }\in \mathbf {\Theta }, v \in W, \end{aligned}$$

39 $$\begin{aligned} \int _\varOmega (\nabla w - \varvec{\theta }) \cdot \varvec{\delta }\, dx + \mu ^{-1} t^2 \int _\varOmega \varvec{\gamma }\cdot \varvec{\delta }\, dx&= 0&\forall \varvec{\delta }\in \mathbf {\Gamma }. \end{aligned}$$

We observe that for system (37)–(39) also the limit case of $t=0$ is well-defined, where the term $\mu ^{-1} t^2 \int _\varOmega \varvec{\gamma }\cdot \varvec{\delta }\, dx$ vanishes. We reorder terms to obtain a mixed problem in the spirit of [13]. We introduce bilinear forms $a_t: (\underline{\mathbf {M}}\times \mathbf {\Gamma })\times (\underline{\mathbf {M}}\times \mathbf {\Gamma }) \rightarrow \mathbb R$ depending on *t* and $b: (\underline{\mathbf {M}}\times \mathbf {\Gamma })\times (\mathbf {\Theta }\times W) \rightarrow \mathbb R$ independent of *t*

40 $$\begin{aligned} a_t(\underline{\mathbf {m}}, \varvec{\gamma }; \underline{\varvec{\tau }}, \varvec{\delta })= & {} \int _\varOmega \underline{\mathbf {A}}_b\underline{\mathbf {m}}: \underline{\varvec{\tau }}\, dx + \mu ^{-1} t^2 \int _\varOmega \varvec{\gamma }\cdot \varvec{\delta }\, dx , \end{aligned}$$

41 $$\begin{aligned} b(\underline{\mathbf {m}}, \varvec{\gamma }; \varvec{\theta }, w)= & {} \langle \mathbf {{div}}\underline{\mathbf {m}}, \varvec{\theta }\rangle + \int _\varOmega \varvec{\gamma }\cdot (\nabla w - \varvec{\theta })\,dx. \end{aligned}$$

From (37), (38), (39) we obtain a saddle point problem of finding $\underline{\mathbf {m}}\in \underline{\mathbf {M}}$, $\varvec{\gamma }\in \mathbf {\Gamma }$, $\varvec{\theta }\in \mathbf {\Theta }$ and $w \in W$ such that

42 $$\begin{aligned} a_t(\underline{\mathbf {m}}, \varvec{\gamma }; \underline{\varvec{\tau }}, \varvec{\delta }) + b(\underline{\varvec{\tau }}, \varvec{\delta }; \varvec{\theta }, w)&= 0&\forall \underline{\varvec{\tau }}\in \underline{\mathbf {M}}, \varvec{\delta }\in \mathbf {\Gamma } \end{aligned}$$

43 $$\begin{aligned} b(\underline{\mathbf {m}}, \varvec{\gamma }; \varvec{\eta }, v)&= -\int _\varOmega gv\,dx&\forall \varvec{\eta }\in \mathbf {\Theta }, v \in W. \end{aligned}$$

In the limiting case of an infinitely thin (Kirchhoff) plate with $t=0$, it is well known (see e.g. [13, Proposition 10.4.3]) that for $t\rightarrow 0$ the shear $\varvec{\gamma }$ stays bounded in $\mathbf {\Gamma }_0 := {\mathbf {H}^{-1}({\text {div}})}$. We will see that $\mathbf {\Gamma }_0$ is the natural space for the analysis of the case $t=0$. Note that $a_0(\cdot ,\cdot )$ is well-defined on $\underline{\mathbf {M}}\times \mathbf {\Gamma }_0$, while for $t>0$ $a_t(\cdot ,\cdot )$, cannot be evaluated on the whole space $\underline{\mathbf {M}}\times \mathbf {\Gamma }_0$. The bilinear form $b(\cdot ,\cdot )$ is also well-defined in the limiting case, as we shall show below.

For the stability analysis of (42), (43) by Brezzi’s theory [13, Theorem 4.2.3] a characterization of the kernel space ${\text {Ker}}(B)$ is needed, which is provided in the following lemma.

### Lemma 1

Define the kernel space

44 $$\begin{aligned} {\text {Ker}}(B) := \left\{ (\underline{\mathbf {m}},\varvec{\gamma }) \in \underline{\mathbf {M}}\times \mathbf {\Gamma }:\ b(\underline{\mathbf {m}},\varvec{\gamma };\varvec{\eta },v) = 0 \ \text{ for } \text{ all }\ (\varvec{\eta },v)\in \mathbf {\Theta }\times W\right\} . \end{aligned}$$

Then any $(\underline{\mathbf {m}},\varvec{\gamma }) \in {\text {Ker}}(B)$ satisfies

45 $$\begin{aligned} \langle {\text {div}}\underline{\mathbf {m}}, \nabla v\rangle = 0 \qquad \forall v \in H^1_0 \end{aligned}$$

and

46 $$\begin{aligned} \langle {\text {div}}\underline{\mathbf {m}}- \varvec{\gamma }, \varvec{\eta }\rangle = 0 \qquad \forall \varvec{\eta }\in {\mathbf {H}}_0({\text {curl}}). \end{aligned}$$

These equalities also hold when $\mathbf {\Gamma }= {\mathbf {L}}^2$ is replaced by $\mathbf {\Gamma }_0 = {\mathbf {H}}^{-1}({\text {div}})$.

### Proof

The proof follows directly, setting either $\varvec{\eta }= \nabla v$ or $v=0$ in (44). Note that all duality products are well-defined due to the choice of spaces. $\square$

### The limiting case $t=0$

We prove existence and uniqueness of the solution for the limiting case of an infinitely thin (Kirchhoff) plate with $t=0$. As mentioned in the introduction, this case is closely related to the Hellan–Herrmann–Johnson formulation, when setting $\varvec{\theta }= \nabla w$ and eliminating thereby the unknowns $\varvec{\theta }$ and $\varvec{\gamma }$. However, we will present an analysis of the full mixed system, as it will help understand the case of small thickness $t > 0$.

As already mentioned, in this case the natural choice for the shear space is $\mathbf {\Gamma }_0 := {\mathbf {H}^{-1}({\text {div}})}$. We show boundedness and stability estimates for the bilinear forms, which implies existence, uniqueness and stability of the solution [13, Theorem 4.2.3]. We use the following natural norms

47 $$\begin{aligned} \Vert \underline{\mathbf {m}}, \varvec{\gamma }\Vert _{\underline{\mathbf {M}}\times \mathbf {\Gamma }_0}^2:= & {} \Vert \underline{\mathbf {m}}\Vert _{{\underline{\mathbf {H}}({\text {div}}\,\mathbf {{div}})}}^2 + \Vert \varvec{\gamma }\Vert _{{\mathbf {H}}^{-1}({\text {div}})}^2, \end{aligned}$$

48 $$\begin{aligned} \Vert \varvec{\theta }, w\Vert _{\mathbf {\Theta }\times W}:= & {} \Vert \varvec{\theta }\Vert _{{\mathbf {H}({\text {curl}})}}^2 + \Vert w\Vert _{H^1}^2. \end{aligned}$$

#### Lemma 2

The bilinear form $a_0: (\underline{\mathbf {M}}\times \mathbf {\Gamma }_0)\times (\underline{\mathbf {M}}\times \mathbf {\Gamma }_0)$ is bounded, for all $\underline{\mathbf {m}},\underline{\varvec{\tau }}\in \underline{\mathbf {M}}$ and $\varvec{\gamma },\varvec{\delta }\in \mathbf {\Gamma }_0$

49 $$\begin{aligned} a_0(\underline{\mathbf {m}},\varvec{\gamma };\underline{\varvec{\tau }},\varvec{\delta }) \le \bar{\alpha }_0 \Vert \underline{\mathbf {m}},\varvec{\gamma }\Vert _{\underline{\mathbf {M}}\times \mathbf {\Gamma }_0}\Vert \underline{\varvec{\tau }},\varvec{\delta }\Vert _{\underline{\mathbf {M}}\times \mathbf {\Gamma }_0}. \end{aligned}$$

Moreover, it is coercive on ${\text {Ker}}(B)$, for all $(\underline{\mathbf {m}},\varvec{\gamma }) \in {\text {Ker}}(B)$

50 $$\begin{aligned} a_0(\underline{\mathbf {m}},\varvec{\gamma };\underline{\mathbf {m}},\varvec{\gamma }) \ge \underline{\alpha }_0 \Vert \underline{\mathbf {m}},\varvec{\gamma }\Vert _{\underline{\mathbf {M}}\times \mathbf {\Gamma }_0}^2. \end{aligned}$$

#### Proof

Boundedness of $a_0$ is straightforward, since $\underline{\mathbf {M}}\subset {\underline{\mathbf {L}}}^2$, and

51 $$\begin{aligned} a_0(\underline{\mathbf {m}},\varvec{\gamma };\underline{\varvec{\tau }},\varvec{\delta }) \le \lambda _{max}(\underline{\mathbf {A}}_b)\Vert \underline{\mathbf {m}}\Vert _{{\underline{\mathbf {L}}}^2}^2 \le \lambda _{max}(\underline{\mathbf {A}}_b)\Vert \underline{\mathbf {m}},\varvec{\gamma }\Vert _{\underline{\mathbf {M}}\times \mathbf {\Gamma }_0}. \end{aligned}$$

We proceed to showing coercivity of $a_0$ on ${\text {Ker}}(B)$. We use Eqs. (45) and (46) of Lemma 1 to bound $\varvec{\gamma }$ by $\underline{\mathbf {m}}$ in their respective norms. Note that for $\mathbf {v}\in {\mathbf {H}}^1_0$ and $w \in H^1_0$ we have $\mathbf {v}\in {\mathbf {H}}_0({\text {curl}})$ and $\nabla w \in {\mathbf {H}}_0({\text {curl}})$.

52 $$\begin{aligned} \Vert \varvec{\gamma }\Vert _{{\mathbf {H}^{-1}({\text {div}})}}^2&\mathop {=}\limits ^{(8)}&\sup _{\mathbf {v}\in {\mathbf {H}}^1_0} \frac{\langle \varvec{\gamma },\mathbf {v}\rangle ^2}{\Vert \nabla \mathbf {v}\Vert _{{\underline{\mathbf {L}}}^2}^2} + \sup _{w \in H^1_0} \frac{\langle \varvec{\gamma }, \nabla w\rangle ^2}{\Vert \nabla w\Vert _{{\mathbf {L}}^2}^2} \end{aligned}$$

53 $$\begin{aligned}&\mathop {=}\limits ^{(46)}&\sup _{\mathbf {v}\in {\mathbf {H}}^1_0} \frac{\langle \mathbf {{div}}\underline{\mathbf {m}},\mathbf {v}\rangle ^2}{\Vert \nabla \mathbf {v}\Vert _{{\underline{\mathbf {L}}}^2}^2} + \sup _{w \in H^1_0} \frac{\langle \mathbf {{div}}\underline{\mathbf {m}}, \nabla w\rangle ^2}{\Vert \nabla w\Vert _{{\mathbf {L}}^2}^2} \end{aligned}$$

54 $$\begin{aligned}&\mathop {=}\limits ^{(17),(45)}&\sup _{\mathbf {v}\in {\mathbf {H}}^1_0} \frac{\left( -\int _\varOmega \underline{\mathbf {m}}:\underline{\varvec{\varepsilon }}(\mathbf {v})\,dx+0\right) ^2}{\Vert \nabla \mathbf {v}\Vert _{{\underline{\mathbf {L}}}^2}^2} + 0 \end{aligned}$$

55 $$\begin{aligned}\le & {} \Vert \underline{\mathbf {m}}\Vert _{{\underline{\mathbf {L}}}^2}^2. \end{aligned}$$

Now coercivity of $a_0$ is ensured by the minimal eigenvalue $\lambda _{min}(\underline{\mathbf {A}}_b)$ of the compliance tensor $\underline{\mathbf {A}}_b$,

56 $$\begin{aligned} a_0(\underline{\mathbf {m}},\varvec{\gamma }; \underline{\mathbf {m}},\varvec{\gamma })= & {} \int _{\varOmega }(\underline{\mathbf {A}}_b\underline{\mathbf {m}}):\underline{\mathbf {m}}\,dx \end{aligned}$$

57 $$\begin{aligned}\ge & {} \lambda _{min}(\underline{\mathbf {A}}_b)\Vert \underline{\mathbf {m}}\Vert _{{\underline{\mathbf {L}}}^2}^2 + \underbrace{\sup _{w \in H^1_0} \frac{\langle \mathbf {{div}}\underline{\mathbf {m}}, \nabla w\rangle ^2}{\Vert \nabla w\Vert _{{\mathbf {L}}^2}^2}}_{=0\ \mathrm {by}\ (46)} \end{aligned}$$

58 $$\begin{aligned}\ge & {} \frac{1}{2}\lambda _{min}(\underline{\mathbf {A}}_b)\Vert \underline{\mathbf {m}}, \varvec{\gamma }\Vert _{\underline{\mathbf {M}}\times \mathbf {\Gamma }_0}^2. \end{aligned}$$

$$\square $$

#### Lemma 3

The bilinear form $b: (\underline{\mathbf {M}}\times \mathbf {\Gamma }_0)\times (\mathbf {\Theta }\times W)$ is bounded, for all $\underline{\varvec{\tau }}\in \underline{\mathbf {M}}$, $\varvec{\delta }\in \mathbf {\Gamma }_0$, $\varvec{\eta }\in \mathbf {\Theta }$ and $v \in W$

59 $$\begin{aligned} b(\underline{\varvec{\tau }},\varvec{\delta }; \varvec{\eta },v) \le \bar{\beta }_0 \Vert \underline{\varvec{\tau }},\varvec{\delta }\Vert _{\underline{\mathbf {M}}\times \mathbf {\Gamma }_0}\Vert \varvec{\eta },v\Vert _{\mathbf {\Theta }\times W}. \end{aligned}$$

Moreover, it is satisfies an inf-sup condition, for all $\varvec{\theta }\in \mathbf {\Theta }$, $w\in W$ there exist $\underline{\mathbf {m}}\in \underline{\mathbf {M}}$, $\varvec{\gamma }\in \mathbf {\Gamma }$ such that

60 $$\begin{aligned} b(\underline{\mathbf {m}},\varvec{\gamma };\varvec{\theta },w) \ge \underline{\beta }_0 \Vert \underline{\mathbf {m}},\varvec{\gamma }\Vert _{\underline{\mathbf {M}}\times \mathbf {\Gamma }_0} \Vert \varvec{\theta },w\Vert _{\mathbf {\Theta }\times W}. \end{aligned}$$

#### Proof

Boundedness follows directly by the choice of spaces. We proceed to show the inf-sup condition. Let $\varvec{\theta }\in \mathbf {\Theta }, w \in W$ be fixed.

From [40] we know that $\langle \mathbf {{div}}\underline{\mathbf {m}}, \varvec{\theta }\rangle$ is inf-sup stable on ${\underline{\mathbf {H}}({\text {div}}\,\mathbf {{div}})}\times {\mathbf {H}_0({\text {curl}})}$, i.e. there exists some ${\tilde{\underline{\mathbf {m}}}} \in {\underline{\mathbf {H}}({\text {div}}\,\mathbf {{div}})}$ such that

61 $$\begin{aligned} \langle \mathbf {{div}}{\tilde{\underline{\mathbf {m}}}}, \varvec{\theta }\rangle \ge c_1 \Vert {\tilde{\underline{\mathbf {m}}}}\Vert _{{\underline{\mathbf {H}}({\text {div}}\,\mathbf {{div}})}} \Vert \varvec{\theta }\Vert _{{\mathbf {H}({\text {curl}})}} \end{aligned}$$

and we have shown

62 $$\begin{aligned} \underline{c}\Vert \varvec{\theta }\Vert _{{\mathbf {H}({\text {curl}})}} \le \Vert \tilde{\underline{\mathbf {m}}}\Vert _{{\underline{\mathbf {H}}({\text {div}}\,\mathbf {{div}})}} \le \overline{c} \Vert \varvec{\theta }\Vert _{{\mathbf {H}({\text {curl}})}}, \end{aligned}$$

with $c_1 > 0$ and $0 < \underline{c} \le 1 \le \overline{c}$. Moreover, since ${\mathbf {H}^{-1}({\text {div}})}= \mathbf {\Gamma }_0$ is the dual space of ${\mathbf {H}_0({\text {curl}})}= \mathbf {\Theta }$, and since $\nabla W\subset \mathbf {\Theta }$, by the Riesz Isomorphism there exists some $\tilde{\varvec{\gamma }} \in \mathbf {\Gamma }_0$ such that

63 $$\begin{aligned} \Vert {\tilde{\varvec{\gamma }}}\Vert _{{\mathbf {H}^{-1}({\text {div}})}} = \Vert \nabla w - \varvec{\theta }\Vert _{{\mathbf {H}({\text {curl}})}} \end{aligned}$$

and

64 $$\begin{aligned} \langle {\tilde{\varvec{\gamma }}}, \nabla w - \varvec{\theta }\rangle = \Vert \nabla w - \varvec{\theta }\Vert _{{\mathbf {H}({\text {curl}})}}^2. \end{aligned}$$

In the remainder of the proof we verify that the pair $(\underline{\mathbf {m}},\varvec{\gamma }) := (\frac{2}{c_1\underline{c}}{\tilde{\underline{\mathbf {m}}}}, {\tilde{\varvec{\gamma }}})$ satisfies the inf-sup condition (60) with stability constant $\underline{\beta }_0 = \frac{c_1\underline{c}}{\sqrt{2}\overline{c}}$. First, we observe

65 $$\begin{aligned} b(\underline{\mathbf {m}}, \varvec{\gamma }; \varvec{\theta }, w)= & {} \langle \mathbf {{div}}\underline{\mathbf {m}}, \varvec{\theta }\rangle + \langle \varvec{\gamma }, \nabla w - \varvec{\theta }\rangle \end{aligned}$$

66 $$\begin{aligned}\ge & {} c_1 \Vert \underline{\mathbf {m}}\Vert _{{\underline{\mathbf {H}}({\text {div}}\,\mathbf {{div}})}}\Vert \varvec{\theta }\Vert _{{\mathbf {H}({\text {curl}})}} + \Vert \varvec{\gamma }\Vert _{{\mathbf {H}^{-1}({\text {div}})}}\Vert \nabla w - \varvec{\theta }\Vert _{{\mathbf {H}({\text {curl}})}} \quad \end{aligned}$$

67 $$\begin{aligned}\ge & {} 2 \Vert \varvec{\theta }\Vert _{{\mathbf {H}({\text {curl}})}}^2 + \Vert \nabla w - \varvec{\theta }\Vert _{{\mathbf {H}({\text {curl}})}}^2. \end{aligned}$$

Using the triangle inequality in the form that $\Vert \varvec{\theta }\Vert _{{\mathbf {H}({\text {curl}})}}^2 + \Vert \nabla w - \varvec{\theta }\Vert _{{\mathbf {H}({\text {curl}})}}^2 \ge \frac{1}{2} \Vert \nabla w\Vert _{{\mathbf {H}({\text {curl}})}}^2$, and the fact that $\Vert \nabla w\Vert _{{\mathbf {H}({\text {curl}})}} = \Vert \nabla w\Vert _{{\mathbf {L}}^2}$, we obtain

68 $$\begin{aligned}&b(\underline{\mathbf {m}}, \varvec{\gamma }; \varvec{\theta }, w) \end{aligned}$$

69 $$\begin{aligned}&\quad \ge \left( 2\Vert \varvec{\theta }\Vert _{{\mathbf {H}({\text {curl}})}}^2 + \Vert \nabla w - \varvec{\theta }\Vert _{{\mathbf {H}({\text {curl}})}}^2\right) ^{1/2} \left( \Vert \varvec{\theta }\Vert _{{\mathbf {H}({\text {curl}})}}^2 + \frac{1}{2}\Vert \nabla w\Vert _{{\mathbf {L}}^2}^2\right) ^{1/2}.\qquad \end{aligned}$$

Since $2\Vert \varvec{\theta }\Vert _{{\mathbf {H}({\text {curl}})}} \ge \frac{2}{\overline{c}}\Vert {\tilde{\underline{\mathbf {m}}}}\Vert _{{\underline{\mathbf {H}}({\text {div}}\,\mathbf {{div}})}} = \frac{c_1\underline{c}}{\overline{c}} \Vert \underline{\mathbf {m}}\Vert _{{\underline{\mathbf {H}}({\text {div}}\,\mathbf {{div}})}}$ due to equation (62) and the definition of $\underline{\mathbf {m}}$, and $\Vert \varvec{\gamma }\Vert _{H^{-1}({\text {div}})} = \Vert \nabla w - \varvec{\theta }\Vert _{H({\text {curl}})}$ due to equation (63), we conclude

70 $$\begin{aligned}&b(\underline{\mathbf {m}}, \varvec{\gamma }; \varvec{\theta }, w) \end{aligned}$$

71 $$\begin{aligned}&\quad \ge \frac{c_1\underline{c}}{\overline{c}}\left( \Vert \underline{\mathbf {m}}\Vert _{{\underline{\mathbf {H}}({\text {div}}\,\mathbf {{div}})}}^2 + \Vert \varvec{\gamma }\Vert _{{\mathbf {H}^{-1}({\text {div}})}}^2 \right) ^{1/2} \left( \Vert \varvec{\theta }\Vert _{{\mathbf {H}({\text {curl}})}}^2 + \frac{1}{2}\Vert \nabla w\Vert _{{\mathbf {L}}^2}^2\right) ^{1/2} \qquad \end{aligned}$$

72 $$\begin{aligned}&\quad = \frac{c_1\underline{c}}{\sqrt{2}\overline{c}} \Vert \underline{\mathbf {m}},\varvec{\gamma }\Vert _{\underline{\mathbf {M}}\times \mathbf {\Gamma }_0} \Vert \varvec{\theta },w\Vert _{\mathbf {\Theta }\times W}. \end{aligned}$$

$$\square $$

#### Theorem 1

For $t=0$, problem (42), (43) has a unique solution $\underline{\mathbf {m}}\in \underline{\mathbf {M}}$, $w \in W$, $\varvec{\theta }\in \mathbf {\Theta }$ and $\varvec{\gamma }\in \mathbf {\Gamma }_0 = {\mathbf {H}^{-1}({\text {div}})}$. The solution is bounded by

73 $$\begin{aligned} \Vert \underline{\mathbf {m}}\Vert _{{\underline{\mathbf {H}}({\text {div}}\,\mathbf {{div}})}} + \Vert \varvec{\gamma }\Vert _{{\mathbf {H}^{-1}({\text {div}})}} + \Vert \varvec{\theta }\Vert _{{\mathbf {H}({\text {curl}})}} + \Vert w\Vert _{H^1} \le c \Vert g\Vert _{H^{-1}} \end{aligned}$$

with *c* a generic constant.

### The case of positive thickness $t>0$

In this section, we prove existence and uniqueness of a solution to the Reissner–Mindlin problem (37)–(39) in the case of positive thickness $t>0$. To this end, a different set of norms is introduced, that includes the thickness *t*.

74 $$\begin{aligned} \Vert \underline{\mathbf {m}}, \varvec{\gamma }\Vert _{\underline{\mathbf {M}}\times \mathbf {\Gamma },t}^2:= & {} \Vert \underline{\mathbf {m}}\Vert _{{\underline{\mathbf {H}}({\text {div}}\,\mathbf {{div}})}}^2 + t\Vert \varvec{\gamma }\Vert _{{\mathbf {L}}^2}^2, \end{aligned}$$

75 $$\begin{aligned} \Vert \varvec{\theta }, w\Vert _{\mathbf {\Theta }\times W,t}^2:= & {} \Vert \varvec{\theta }\Vert _{{\mathbf {H}({\text {curl}})}}^2 + \Vert w\Vert _{H^1}^2 + t^{-2}\Vert \nabla w - \varvec{\theta }\Vert _{{\mathbf {L}}^2}^2. \end{aligned}$$

#### Lemma 4

A norm equivalent to $\Vert \varvec{\theta }, w\Vert _{\mathbf {\Theta }\times W}$ can be defined omitting the term $\Vert w\Vert _{H^1}$ in (75), where the non-trivial bound is characterized by the Friedrichs constant $c_F$,

76 $$\begin{aligned} \Vert \varvec{\theta }, w\Vert _{\mathbf {\Theta }\times W,t}^2 \le \left( 1+2\left( 1+c_F^2\right) \right) \left( \Vert \varvec{\theta }\Vert _{{\mathbf {H}({\text {curl}})}}^2 + t^{-2}\Vert \nabla w - \varvec{\theta }\Vert _{{\mathbf {L}}^2}^2\right) . \end{aligned}$$

#### Proof

The statement of the lemma is clear from the following consideration, which uses Friedrichs’ inequality, the triangle inequality and the fact that $t<1$.

77 $$\begin{aligned} \frac{1}{1+c_F^2}\Vert w\Vert _{H^1}^2\le & {} \Vert \nabla w\Vert _{{\mathbf {L}}^2}^2 \le 2\left( \Vert \varvec{\theta }\Vert _{{\mathbf {L}}^2}^2 + \Vert \nabla w -\varvec{\theta }\Vert _{{\mathbf {L}}^2}^2\right) \end{aligned}$$

78 $$\begin{aligned}\le & {} 2\left( \Vert \varvec{\theta }\Vert _{{\mathbf {H}({\text {curl}})}}^2 + t^{-2}\Vert \nabla w-\varvec{\theta }\Vert _{{\mathbf {L}}^2}^2\right) . \end{aligned}$$

$$\square $$

The next two lemmas provide stability estimates for the bilinear forms in the *t*-dependent norms $\Vert \underline{\mathbf {m}},\varvec{\gamma }\Vert _{\underline{\mathbf {M}}\times \mathbf {\Gamma },t}$ and $\Vert \varvec{\theta },w\Vert _{\mathbf {\Theta }\times W,t}$. These estimates are used to ensure existence and uniqueness of a solution and to obtain a stability estimate not deteriorating with $t \rightarrow 0$. The proof of Lemma 5 is straightforward in the manner of the proof of Lemma 2:

#### Lemma 5

For $t>0$, the bilinear form $a_t: (\underline{\mathbf {M}}\times \mathbf {\Gamma })\times (\underline{\mathbf {M}}\times \mathbf {\Gamma })$ is bounded, for all $\underline{\mathbf {m}},\underline{\varvec{\tau }}\in \underline{\mathbf {M}}$ and $\varvec{\gamma },\varvec{\delta }\in \mathbf {\Gamma }$

79 $$\begin{aligned} a_t(\underline{\mathbf {m}},\varvec{\gamma };\underline{\varvec{\tau }},\varvec{\delta }) \le \bar{\alpha }\Vert \underline{\mathbf {m}},\varvec{\gamma }\Vert _{\underline{\mathbf {M}}\times \mathbf {\Gamma },t}\Vert \underline{\varvec{\tau }},\varvec{\delta }\Vert _{\underline{\mathbf {M}}\times \mathbf {\Gamma },t}. \end{aligned}$$

It is coercive on ${\text {Ker}}(B)$, for all $(\underline{\mathbf {m}},\varvec{\gamma }) \in {\text {Ker}}(B)$

80 $$\begin{aligned} a_t(\underline{\mathbf {m}},\varvec{\gamma };\underline{\mathbf {m}},\varvec{\gamma }) \ge \underline{\alpha } \Vert \underline{\mathbf {m}},\varvec{\gamma }\Vert _{\underline{\mathbf {M}}\times \mathbf {\Gamma },t}^2. \end{aligned}$$

The constants $\bar{\alpha }$, $\underline{\alpha }$ are independent of the thickness *t*.

#### Lemma 6

The bilinear form $b: (\underline{\mathbf {M}}\times \mathbf {\Gamma })\times (\mathbf {\Theta }\times W)$ is continuous with respect to the *t*-dependent norms, for all $\underline{\varvec{\tau }}\in \underline{\mathbf {M}}$, $\varvec{\delta }\in \mathbf {\Gamma }$, $\varvec{\eta }\in \mathbf {\Theta }$ and $v \in W$

81 $$\begin{aligned} b(\underline{\varvec{\tau }},\varvec{\delta }; \varvec{\eta },v) \le \bar{\beta }\Vert \underline{\varvec{\tau }},\varvec{\delta }\Vert _{\underline{\mathbf {M}}\times \mathbf {\Gamma },t}\Vert \varvec{\eta },v\Vert _{\mathbf {\Theta }\times W,t}. \end{aligned}$$

Moreover, it is satisfies an inf-sup condition, for all $\varvec{\theta }\in \mathbf {\Theta }$, $w\in W$ there exist $\underline{\mathbf {m}}\in \underline{\mathbf {M}}$, $\varvec{\gamma }\in \mathbf {\Gamma }$ such that

82 $$\begin{aligned} b(\underline{\mathbf {m}},\varvec{\gamma };\varvec{\theta },w) \ge \underline{\beta } \Vert \underline{\mathbf {m}},\varvec{\gamma }\Vert _{\underline{\mathbf {M}}\times \mathbf {\Gamma },t} \Vert \varvec{\theta },w\Vert _{\mathbf {\Theta }\times W,t}. \end{aligned}$$

The constants $\bar{\beta }$, $\underline{\beta }$ are independent of the thickness *t*.

#### Proof

Obviously, the bilinear form is bounded, as the divergence term is bounded in ${\underline{\mathbf {H}}}({\text {div}}\mathbf {{div}}) \times {\mathbf {H}}({\text {curl}})$, and the integral is bounded by the respective scaled ${\mathbf {L}}^2$-norms $t\Vert \varvec{\delta }\Vert _{{\mathbf {L}}^2}$ and $t^{-1}\Vert \nabla w - \theta \Vert _{{\mathbf {L}}^2}$.

To prove the inf-sup condition, assume $\varvec{\theta }\in \mathbf {\Theta }$ and $w \in W$ are given. Similar to the proof of Lemma 3, we use the theory provided in [40]. We choose $\underline{\mathbf {m}}={\tilde{\underline{\mathbf {m}}}} \in \underline{\mathbf {M}}= {\underline{\mathbf {H}}({\text {div}}\,\mathbf {{div}})}$ from Eqs. (61) and (62). Additionally, we choose $\varvec{\gamma }= t^{-2}(\nabla w - \varvec{\theta })$. This is possible since $\nabla w - \varvec{\theta }\in {\mathbf {H}({\text {curl}})}\subset \mathbf {L}^2$. Then we have

83 $$\begin{aligned} \int _{\varOmega } \varvec{\gamma }\cdot ( \nabla w - \varvec{\theta })\,dx = t^{-2}\Vert \nabla w - \varvec{\theta }\Vert _{{\mathbf {L}}^2}^2. \end{aligned}$$

Combining the definition of $b(\cdot ,\cdot )$ (41), Eq. (83) and the bounds from (61) and (62) we obtain

84 $$\begin{aligned} b(\underline{\mathbf {m}},\varvec{\gamma };\varvec{\theta },w)= & {} \langle \mathbf {{div}}\underline{\mathbf {m}}, \varvec{\theta }\rangle + \int _{\varOmega }\varvec{\gamma }\cdot ( \nabla w - \varvec{\theta })\,dx \end{aligned}$$

85 $$\begin{aligned}\ge & {} c_1\underline{c}\Vert \varvec{\theta }\Vert _{{\mathbf {H}({\text {curl}})}}^2 + t^{-2}\Vert \nabla w - \varvec{\theta }\Vert _{{\mathbf {L}}^2}^2. \end{aligned}$$

Basic algebra for real numbers, the definition of the shear $\varvec{\gamma }$ and the upper bound in (62) lead to

86 $$\begin{aligned}&b(\underline{\mathbf {m}},\varvec{\gamma };\varvec{\theta },w) \end{aligned}$$

87 $$\begin{aligned}&\ge c_1 \underline{c} \big (\Vert \varvec{\theta }\Vert _{{\mathbf {H}({\text {curl}})}}^2 + t^{-2}\Vert \nabla w - \varvec{\theta }\Vert _{{\mathbf {L}}^2}^2\big ) \end{aligned}$$

88 $$\begin{aligned}&\ge \frac{c_1 \underline{c}}{\overline{c}} \left( \Vert \underline{\mathbf {m}}\Vert _{{\underline{\mathbf {H}}({\text {div}}\,\mathbf {{div}})}}^2+t^2\Vert \varvec{\gamma }\Vert _{{\mathbf {L}}^2}^2\right) ^{1/2} \big (\Vert \varvec{\theta }\Vert _{{\mathbf {H}({\text {curl}})}}^2 + t^{-2}\Vert \nabla w - \varvec{\theta }\Vert _{{\mathbf {L}}^2}^2\big )^{1/2}. \end{aligned}$$

We see inf-sup stability for $b(\cdot ,\cdot )$ using the bound from Lemma 4

89 $$\begin{aligned} b(\underline{\mathbf {m}},\varvec{\gamma };\varvec{\theta },w)\ge & {} \frac{c_1 \underline{c}}{\overline{c}\sqrt{1+2(1+c_F^2)}} \Vert \underline{\mathbf {m}},\varvec{\gamma }\Vert _{\underline{\mathbf {M}}\times \mathbf {\Gamma },t} \Vert \varvec{\theta },w\Vert _{\mathbf {\Theta }\times W,t}. \end{aligned}$$

$$\square $$

#### Theorem 2

For $t>0$, problem (42), (43) has a unique solution $\underline{\mathbf {m}}\in \underline{\mathbf {M}}$, $w \in W$, $\varvec{\theta }\in \mathbf {\Theta }$ and $\varvec{\gamma }\in \mathbf {\Gamma }$, which is bounded as below

90 $$\begin{aligned} \Vert \underline{\mathbf {m}}\Vert _{{\underline{\mathbf {H}}({\text {div}}\,\mathbf {{div}})}} + \Vert \varvec{\gamma }\Vert _{{\mathbf {H}}^{-1}({\text {div}})} + t\Vert \varvec{\gamma }\Vert _{{\mathbf {L}}^2} + \Vert \varvec{\theta }\Vert _{{\mathbf {H}({\text {curl}})}} + \Vert w\Vert _{H^1} \le c \Vert g\Vert _{H^{-1}} \end{aligned}$$

where *c* is a generic constant independent of *t*.

#### Proof

Again, we use the statement of [13, Theorem 4.2.3], where coercivity (Lemma 5) and inf-sup stability (Lemma 6) ensure the existence and stability of a unique solution. Note that, for the solution $\underline{\mathbf {m}}\in \underline{\mathbf {M}}$, $w \in W$, $\varvec{\theta }\in \mathbf {\Theta }$ and $\varvec{\gamma }\in \mathbf {\Gamma }$, there holds $\varvec{\gamma }= \mu t^{-2} (\nabla w - \varvec{\theta })$. Thus $t\Vert \varvec{\gamma }\Vert _{{\mathbf {L}}^2} = t^{-1}\Vert \nabla w - \varvec{\theta }\Vert _{{\mathbf {L}}^2}$, which ensures the estimate

91 $$\begin{aligned} \Vert \underline{\mathbf {m}}\Vert _{{\underline{\mathbf {H}}({\text {div}}\,\mathbf {{div}})}} + t\Vert \varvec{\gamma }\Vert _{{\mathbf {L}}^2} + \Vert \varvec{\theta }\Vert _{{\mathbf {H}({\text {curl}})}} + \Vert w\Vert _{H^1} \le c \Vert g\Vert _{H^{-1}}. \end{aligned}$$

We add the bound on $\Vert \varvec{\gamma }\Vert _{{\mathbf {H}}^{-1}({\text {div}})}$: since ${\mathbf {H}}^{-1}({\text {div}})$ is dual to $\mathbf {\Theta }= {\mathbf {H}}({\text {curl}})$ and the solution $\varvec{\gamma }$ satisfies the variational equation (38) with $v = 0$ we have

92 $$\begin{aligned} \Vert \varvec{\gamma }\Vert _{{\mathbf {H}}^{-1}({\text {div}})} = \sup _{\varvec{\eta }\in \mathbf {\Theta }} \frac{\langle \varvec{\gamma }, \varvec{\eta }\rangle }{\Vert \varvec{\eta }\Vert _{\mathbf {\Theta }}} = \sup _{\varvec{\eta }\in \mathbf {\Theta }} \frac{\langle \mathbf {{div}}\underline{\mathbf {m}}, \varvec{\eta }\rangle }{\Vert \varvec{\eta }\Vert _{\mathbf {\Theta }}} \le \Vert \underline{\mathbf {m}}\Vert _{\underline{\mathbf {M}}}. \end{aligned}$$

Thus, the statement of the theorem is shown.$\square$

## Finite elements

Throughout this section, let $\varOmega \subset \mathbb R^2$ be a polygonal Lipschitz domain, and let $(\mathcal {T}_h)$ be a family of decompositions into triangular elements *T*. We assume that the family $(\mathcal {T}_h)$ is regular, shape-regular and quasi-uniform with mesh size *h* (see e.g. [15]). Moreover, $(\mathcal {F}_h)$ shall denote the set of edges in the mesh.

We propose a finite element method without using the additional unknown shear $\varvec{\gamma }$, having the form of (35)–(36). We use the TDNNS finite element spaces for the bending moments and rotations, and a fully continuous Lagrange space for the deflection. For integer $k \ge 1$, $\underline{\mathbf {m}}$ is approximated in the normal–normal continuous space of order *k* introduced in [38], while $\varvec{\theta }$ is discretized by order *k* Nédélec elements of the second kind [37]. The deflection elements are order $k+1$ continuous elements. In detail, we choose

93 $$\begin{aligned} \underline{\mathbf {M}}_h:= & {} \left\{ \underline{\mathbf {m}}_h \in {\underline{\mathbf {L}}^2_{sym}(\varOmega )}: \underline{\mathbf {m}}_h|_T \in P^k, m_{h,nn} \text{ continuous }\right\} , \end{aligned}$$

94 $$\begin{aligned} \mathbf {\Theta }_h:= & {} \left\{ \varvec{\theta }_h \in {\mathbf {L}^2(\varOmega )}: \varvec{\theta }_h|_T \in P^k, \varvec{\theta }_{h,{\varvec{\tau }}} \text{ continuous }, \varvec{\theta }_{h,{\varvec{\tau }}} = 0 \text{ on } \partial \varOmega \right\} , \end{aligned}$$

95 $$\begin{aligned} W_h:= & {} \left\{ w_h \in H^1_0(\varOmega ): w_h|_T \in P^{k+1}\right\} . \end{aligned}$$

The discrete system used in implementations reads

96 $$\begin{aligned} \int _\varOmega \underline{\mathbf {A}}_b\underline{\mathbf {m}}_h : \underline{\varvec{\tau }}_h\,dx + \langle \mathbf {{div}}\underline{\varvec{\tau }}_h, \varvec{\theta }_h\rangle&= 0 \qquad \forall \underline{\varvec{\tau }}_h \in \underline{\mathbf {M}}_h, \end{aligned}$$

97 $$\begin{aligned} \langle \mathbf {{div}}\underline{\mathbf {m}}_h, \varvec{\eta }_h\rangle - \mu t^{-2} \int _\varOmega (\nabla w_h - \varvec{\theta }_h)\cdot (\nabla v_h - \varvec{\eta }_h)\,dx&= -\int _\varOmega g\,v_h\,dx \nonumber \\&\qquad \forall \varvec{\eta }_h \in \mathbf {\Theta }_h, v_h \in W_h.\quad \end{aligned}$$

Note that the finite element space $\underline{\mathbf {M}}_h$ is (slightly) non-conforming, $\underline{\mathbf {M}}_h \not \subset \underline{\mathbf {M}}= {\underline{\mathbf {H}}}({\text {div}}\mathbf {{div}})$. This is due to lacking continuity of $\mathbf {m}_{\mathbf {n}{\varvec{\tau }}}$ at the corner points (in the interior) of each element, see [40, page 13] for a detailed discussion. However, the duality product $\langle \mathbf {{div}}\underline{\varvec{\tau }}_h, \varvec{\theta }_h\rangle$ can now be understood as the $\mathbf {\Theta }_h^* \times \mathbf {\Theta }_h$ duality product, and can be evaluated by the relations (21)–(22). Moreover, the norm $\Vert \cdot \Vert _{\underline{\mathbf {M}}}$ from (18) is not well-defined for $\underline{\mathbf {m}}_h \in \underline{\mathbf {M}}_h$. In [40] we provided a discrete norm and a corresponding stability analysis for the TDNNS continuum mechanics elements. We will use this discrete norm in the current paper, defining

98 $$\begin{aligned} \Vert \underline{\mathbf {m}}\Vert _{\underline{\mathbf {M}}_h}^2 := \Vert \underline{\mathbf {m}}\Vert _{{\underline{\mathbf {L}}}^2}^2 + \sum _{F \in \mathcal E} h_F\Vert m_{nn}\Vert _{L^2(F)}^2 + \sup _{w_h \in W_h} \frac{\langle \mathbf {{div}}\underline{\mathbf {m}}, \nabla w_h\rangle ^2}{\Vert \nabla w_h\Vert _{{\mathbf {L}}^2(\varOmega )}^2}, \end{aligned}$$

and the parameter-dependent norm

99 $$\begin{aligned} \Vert \underline{\mathbf {m}}, \varvec{\gamma }\Vert _{\underline{\mathbf {M}}_h \times \mathbf {\Gamma }_h,t}^2:= & {} \Vert \underline{\mathbf {m}}\Vert _{\underline{\mathbf {M}}_h}^2 + t^2\Vert \varvec{\gamma }\Vert _{L^2}^2. \end{aligned}$$

For finite element tensors $\underline{\mathbf {m}}_h \in \underline{\mathbf {M}}_h$, the edge $L^2$ terms in the norm above can also be omitted, as they are bounded by the domain ${\underline{\mathbf {L}}}^2$ norm. The divergence operator $\mathbf {{div}}: \underline{\mathbf {M}}_h \rightarrow \mathbf {\Theta }_h^*$ is bounded and LBB-stable, see [40]:

### Theorem 3

There exist positive constants $\beta _1, \beta _2 > 0$ such that for any $\underline{\mathbf {m}}_h \in \underline{\mathbf {M}}_h$ and $\varvec{\theta }_h \in \mathbf {\Theta }_h$

100 $$\begin{aligned} \langle \mathbf {{div}}\underline{\mathbf {m}}_h, \varvec{\theta }_h\rangle \le \beta _1 \Vert \underline{\mathbf {m}}_h\Vert _{\underline{\mathbf {M}}_h} \Vert \theta _h\Vert _{{\mathbf {H}}({\text {curl}})}, \end{aligned}$$

and

101 $$\begin{aligned} \inf _{\varvec{\theta }_h \in \mathbf {\Theta }_h} \sup _{\underline{\mathbf {m}}_h \in \underline{\mathbf {M}}_h} \frac{\langle \mathbf {{div}}\underline{\mathbf {m}}_h, \varvec{\theta }_h\rangle }{\Vert \underline{\mathbf {m}}_h\Vert _{\underline{\mathbf {M}}_h} \Vert \varvec{\theta }_h\Vert _{{\mathbf {H}}({\text {curl}})}} \ge \beta _2. \end{aligned}$$

### Discrete stability

For the analysis, it is convenient to introduce a finite element discretization for the shear $\varvec{\gamma }$, which leads to a discrete system equivalent to (96), (97), but which is of the standard saddle point form (42), (43). The equivalence of the discrete systems is due to the inclusion $\nabla W_h \subset \mathbf {\Theta }_h$, and to our choice $\mathbf {\Gamma }_h = \mathbf {\Theta }_h$. Thus, for $w_h \in W_h$, $\varvec{\theta }_h \in \mathbf {\Theta }_h$ and $\varvec{\gamma }_h \in \mathbf {\Gamma }_h$ the discrete variational equation

102 $$\begin{aligned} \int _{\varOmega } (\nabla w_h - \varvec{\theta }_h)\cdot \varvec{\delta }_h\,dx = \mu ^{-1}t^2 \int _{\varOmega } \varvec{\gamma }_h\cdot \varvec{\delta }_h\,dx \qquad \forall \varvec{\delta }_h \in \mathbf {\Gamma }_h \end{aligned}$$

is equivalent to $\varvec{\gamma }_h = \mu t^{-2}(\nabla w_h - \varvec{\theta }_h)$. This implies that $\varvec{\gamma }_h$ can be eliminated, and the smaller system (96), (97) may be used in implementations.

The stability analysis is similar to the analysis of the infinite dimensional problem for positive thickness presented in Sect. 3.2.

#### Lemma 7

The bilinear form $a_t: (\underline{\mathbf {M}}_h \times \mathbf {\Gamma }_h) \times (\underline{\mathbf {M}}_h \times \mathbf {\Gamma }_h)$ is coercive on ${\text {Ker}}(B_h) := \{(\underline{\mathbf {m}}_h, \varvec{\gamma }_h) \in \underline{\mathbf {M}}_h \times \mathbf {\Gamma }_h: b(\underline{\mathbf {m}}_h,\varvec{\gamma }_h;\varvec{\theta }_h,w_h) = 0 \ \forall \varvec{\theta }_h \in \mathbf {\Theta }_h, w_h \in W_h\}$. There exists a constant $\alpha _1 > 0$ independent of *t*, *h* such that

103 $$\begin{aligned} a_t(\underline{\mathbf {m}}_h,\varvec{\gamma }_h;\underline{\mathbf {m}}_h,\varvec{\gamma }_h) \ge \alpha _1 \Vert \underline{\mathbf {m}}_h,\varvec{\gamma }_h\Vert _{\underline{\mathbf {M}}_h \times \mathbf {\Gamma }_h,t}^2. \end{aligned}$$

#### Proof

For any $(\underline{\mathbf {m}}_h, \varvec{\gamma }_h) \in {\text {Ker}}(B_h)$ we have by definition, setting $\varvec{\theta }_h = \nabla w_h$,

104 $$\begin{aligned} \langle \mathbf {{div}}\underline{\mathbf {m}}_h, \nabla w_h \rangle = 0. \end{aligned}$$

Thus, it follows

105 $$\begin{aligned}&a_t(\underline{\mathbf {m}}_h,\varvec{\gamma }_h;\varvec{\theta }_h,w_h) = \int _{\varOmega }(\underline{\mathbf {A}}_b\underline{\mathbf {m}}_h):\underline{\mathbf {m}}_h\,dx + \mu ^{-1} t^2 \int _\varOmega \varvec{\gamma }_h : \varvec{\gamma }_h\,dx \end{aligned}$$

106 $$\begin{aligned}&\ge \lambda _{min}(\underline{\mathbf {A}}_b)\left( \Vert \underline{\mathbf {m}}_h\Vert _{{\underline{\mathbf {L}}}^2}^2 + \underbrace{\sup _{w_h \in W_h} \frac{\langle \mathbf {{div}}\underline{\mathbf {m}}_h,\nabla w_h\rangle ^2}{\Vert \nabla w_h\Vert _{{\mathbf {L}}^2(\varOmega )}^2}}_{=0}\right) + \mu ^{-1} t^2 \Vert \varvec{\gamma }_h\Vert _{{\mathbf {L}}^2(\varOmega )}^2 \end{aligned}$$

107 $$\begin{aligned}&\ge \min (\lambda _{min}(\underline{\mathbf {A}}_b),\mu ^{-1}) \Vert \underline{\mathbf {m}}_h, \varvec{\gamma }_h\Vert _{\underline{\mathbf {M}}_h \times \mathbf {\Gamma }_h,t}^2. \end{aligned}$$

$$\square $$

#### Lemma 8

The bilinear form $b: (\underline{\mathbf {M}}_h \times \mathbf {\Gamma }_h) \times (\mathbf {\Theta }_h \times W_h)$ is bounded and inf-sup stable, for any $\varvec{\theta }_h \in \mathbf {\Theta }_h, w_h \in W_h$ there exist $\underline{\mathbf {m}}_h \in \underline{\mathbf {M}}_h, \varvec{\gamma }_h \in \mathbf {\Gamma }_h$ such that

108 $$\begin{aligned} b(\underline{\mathbf {m}}_h, \varvec{\gamma }_h;\varvec{\theta }_h,w_h) \ge \beta \Vert \underline{\mathbf {m}}_h,\varvec{\gamma }_h\Vert _{\underline{\mathbf {M}}_h\times \mathbf {\Gamma }_h,t} \Vert \varvec{\theta }_h, w_h\Vert _{\mathbf {\Theta }\times W,t}. \end{aligned}$$

Boundedness is clear from the discrete boundedness of the divergence operator, see Theorem 3.

The discrete inf-sup condition is shown in the same manner as in the infinite dimensional case in Theorem 2. The arguments shall not be repeated here, but only shortly commented on.

We use the discrete inf-sup stability of the divergence operator from Theorem 3. As in Theorem 2, we set $\varvec{\gamma }_h = t^{-2}(\nabla w_h - \varvec{\theta }_h)$, which is possible since $\nabla W_h \subset \mathbf {\Theta }_h = \mathbf {\Gamma }_h$. The remainder of the proof involves the same steps as shown in Eqs. (84)–(89), only replacing the infinite-dimensional norms by the discrete ones.

### A-priori error estimates

To get a-priori error estimates, it is necessary to have interpolation error estimates. We use the standard nodal interpolation operator $\mathcal {I}_{W}$ for $H^1$ and the standard interpolator $\mathcal {I}_{\mathbf {\Theta }}$ of the Nédélec space defined using its degrees of freedom, see e.g. [35] for their definition. The following approximation properties for sufficiently smooth functions are provided there for $1 \le m \le k$,

109 $$\begin{aligned} \Vert \varvec{\theta }- \mathcal {I}_{\mathbf {\Theta }}\varvec{\theta }\Vert _{{\mathbf {H}}({\text {curl}})}^2\le & {} c \sum _{T\in \mathcal {T}_h} h^{2m} \Vert \varvec{\theta }\Vert ^2_{{\underline{\mathbf {H}}}^{m+1}(T)} , \end{aligned}$$

110 $$\begin{aligned} \Vert w - \mathcal {I}_{W}w\Vert _{H^1}^2\le & {} c \sum _{T\in \mathcal {T}_h} h^{2m} \Vert w\Vert ^2_{H^{m+1}(T)} . \end{aligned}$$

An important property of the interpolation operators is that they commute with the gradient operator, see e.g. [35, Theorem 5.49],

111 $$\begin{aligned} \mathcal {I}_{\mathbf {\Theta }}\nabla w = \nabla \mathcal {I}_{W}w. \end{aligned}$$

For the bending moments $\underline{\mathbf {m}}$ we use the nodal interpolation operator $\mathcal {I}_{\underline{\mathbf {M}}}$, which is provided and analyzed in [40]. An error estimate in the discrete ${\underline{\mathbf {H}}}({\text {div}}\mathbf {{div}})$ norm was found for $\mathcal {I}_{\underline{\mathbf {M}}}$ for $0 \le l \le k$

112 $$\begin{aligned} \Vert \underline{\mathbf {m}}- \mathcal {I}_{\underline{\mathbf {M}}}\underline{\mathbf {m}}\Vert _{\underline{\mathbf {M}}_h}^2\le & {} c \sum _{T\in \mathcal {T}_h} h^{2(l+1)} \Vert \underline{\mathbf {m}}\Vert ^2_{{\underline{\mathbf {H}}}^{l+1}(T)} . \end{aligned}$$

We shall not provide the degrees of freedom of the stress space in detail here, only note that in two space dimensions there are edge-based degrees of freedom coupling the normal–normal component $m_{nn}$ of $\underline{\mathbf {m}}$, and inner degrees of freedom which can be eliminated by static condensation. Corresponding polynomial basis functions can be found in [38].

We can now venture to show a convergence result of the proposed finite element method.

#### Theorem 4

Let $\underline{\mathbf {m}}\in \underline{\mathbf {M}}$, $w \in W$ and $\varvec{\theta }\in \mathbf {\Theta }$ be the exact solution to the Reissner–Mindlin problem (35), (36), and let $\underline{\mathbf {m}}_h \in \underline{\mathbf {M}}_h$, $w_h \in W_h$ and $\varvec{\theta }_h \in \mathbf {\Theta }_h$ be the corresponding finite element solution. Then we have the a-priori error estimate for $1 \le m \le k$

113 $$\begin{aligned}&\Vert \varvec{\theta }-\varvec{\theta }_h\Vert _{{\mathbf {H}}({\text {curl}})} + \Vert w-w_h\Vert _{H^1(\varOmega )} + \Vert \underline{\mathbf {m}}- \underline{\mathbf {m}}_h\Vert _{\underline{\mathbf {M}}_h} +t\Vert \varvec{\gamma }- \varvec{\gamma }_h\Vert _{{\mathbf {L}}^2(\varOmega )} \end{aligned}$$

114 $$\begin{aligned}&\le c \left( \sum _{T\in \mathcal {T}_h} h^{2m} (\Vert \varvec{\theta }\Vert ^2_{{\mathbf {H}}^{m+1}(T)} + \Vert \underline{\mathbf {m}}\Vert ^2_{{\underline{\mathbf {H}}}^{m}(T)} + t^2\Vert \varvec{\gamma }\Vert _{{\mathbf {H}}^m(T)}^2) \right) ^{1/2}. \end{aligned}$$

#### Proof

Since the finite element method is slightly nonconforming, $\underline{\mathbf {M}}_h \not \subset \underline{\mathbf {M}}$, see [40], we use techniques from Strang’s second lemma. We bound the total error (115) by interpolation error (116) and consistency error (117).

115 $$\begin{aligned} \Vert \varvec{\theta }&- \varvec{\theta }_h\Vert _{{\mathbf {H}}({\text {curl}})} + \Vert w - w_h\Vert _{H^1(\varOmega )} + \Vert \underline{\mathbf {m}}- \underline{\mathbf {m}}_h\Vert _{\underline{\mathbf {M}}_h}+t\Vert \varvec{\gamma }- \varvec{\gamma }_h\Vert _{{\mathbf {L}}^2(\varOmega )} \end{aligned}$$

116 $$\begin{aligned} \le&\left\{ \begin{array}{l} \Vert \varvec{\theta }- \mathcal {I}_{\mathbf {\Theta }}\varvec{\theta }\Vert _{{\mathbf {H}}({\text {curl}})} + \Vert w - \mathcal {I}_{W}w\Vert _{H^1(\varOmega )} + \\ \Vert \underline{\mathbf {m}}- \mathcal {I}_{\underline{\mathbf {M}}}\underline{\mathbf {m}}\Vert _{\underline{\mathbf {M}}_h}+t\Vert \varvec{\gamma }- \mathcal {I}_{\mathbf {\Theta }}\varvec{\gamma }\Vert _{{\mathbf {L}}^2(\varOmega )} \end{array}\right\} \end{aligned}$$

117 $$\begin{aligned}&+\left\{ \begin{array}{l} \Vert \mathcal {I}_{\mathbf {\Theta }}\varvec{\theta }- \varvec{\theta }_h\Vert _{{\mathbf {H}}({\text {curl}})} + \Vert \mathcal {I}_{W}w - w_h\Vert _{H^1(\varOmega )} + \\ \Vert \mathcal {I}_{\underline{\mathbf {M}}}\underline{\mathbf {m}}- \underline{\mathbf {m}}_h\Vert _{\underline{\mathbf {M}}_h}+t\Vert \mathcal {I}_{\mathbf {\Theta }}\varvec{\gamma }- \varvec{\gamma }_h\Vert _{{\mathbf {L}}^2(\varOmega )} \end{array}\right\} \end{aligned}$$

Clearly, the interpolation error (116) can be bounded as stated above (114). We concentrate on the consistency error.

As stated in [13, Theorem 5.2.1], discrete stability ensures

118 $$\begin{aligned}&(117) \end{aligned}$$

119 $$\begin{aligned}&\quad \le \Vert \mathcal {I}_{\mathbf {\Theta }}\varvec{\theta }- \varvec{\theta }_h;\mathcal {I}_{W}w - w_h\Vert _{\mathbf {\Theta }\times W,t} + \Vert \mathcal {I}_{\underline{\mathbf {M}}}\underline{\mathbf {m}}- \underline{\mathbf {m}}_h, \mathcal {I}_{\mathbf {\Theta }}\varvec{\gamma }- \varvec{\gamma }_h\Vert _{\underline{\mathbf {M}}_h\times \mathbf {\Gamma }_h,t}\qquad \quad \end{aligned}$$

120 $$\begin{aligned}&\quad \le \mathop {\mathop {\sup }\limits _{\underline{\varvec{\tau }}_h \in \underline{\mathbf {M}}_h}}\limits _{\varvec{\delta }_h \in \mathbf {\Gamma }_h} \frac{ \left\{ \begin{array}{l} a_t(\mathcal {I}_{\underline{\mathbf {M}}}\underline{\mathbf {m}}- \underline{\mathbf {m}}_h, \mathcal {I}_{\mathbf {\Theta }}\varvec{\gamma }-\varvec{\gamma }_h; \underline{\varvec{\tau }}_h, \varvec{\delta }_h) + \\ b(\underline{\varvec{\tau }}_h,\varvec{\delta }_h; \mathcal {I}_{\mathbf {\Theta }}\varvec{\theta }- \varvec{\theta }_h, \mathcal {I}_{W}w - w_h) \end{array}\right\} }{\Vert \underline{\varvec{\tau }}_h, \varvec{\delta }_h\Vert _{\underline{\mathbf {M}}_h\times \mathbf {\Gamma }_h,t}} \end{aligned}$$

121 $$\begin{aligned}&\qquad + \mathop {\mathop {\sup }\limits _{\varvec{\eta }_h \in \mathbf {\Theta }_h}}\limits _{v_h \in W_h} \frac{b\left( \mathcal {I}_{\underline{\mathbf {M}}}\underline{\mathbf {m}}- \underline{\mathbf {m}}_h, \mathcal {I}_{\mathbf {\Theta }}\varvec{\gamma }- \varvec{\gamma }_h; \nabla v_h - \varvec{\eta }_h\right) }{\Vert \varvec{\eta }_h, v_h\Vert _{\mathbf {\Theta }\times W,t}} \end{aligned}$$

122 $$\begin{aligned}&\quad \le \sup _{\underline{\varvec{\tau }}_h\in \underline{\mathbf {M}}_h} \frac{\int _\varOmega \underline{\mathbf {A}}_b\left( \mathcal {I}_{\underline{\mathbf {M}}}\underline{\mathbf {m}}- \underline{\mathbf {m}}_h\right) :\underline{\varvec{\tau }}_h\,dx + \langle \mathbf {{div}}\underline{\varvec{\tau }}_h, \mathcal {I}_{\mathbf {\Theta }}\varvec{\theta }- \varvec{\theta }_h\rangle }{\Vert \underline{\varvec{\tau }}_h\Vert _{\underline{\mathbf {M}}_h}} \end{aligned}$$

123 $$\begin{aligned}&\qquad +\sup _{\varvec{\delta }_h\in \mathbf {\Gamma }_h} \frac{\int _\varOmega \left( \nabla \mathcal {I}_{W}w-\nabla w_h - \mathcal {I}_{\mathbf {\Theta }}\varvec{\theta }+ \varvec{\theta }_h + \frac{t^2}{\mu }\left( \mathcal {I}_{\mathbf {\Theta }}\varvec{\gamma }-\varvec{\gamma }_h \right) \right) \cdot \varvec{\delta }_h\,dx }{t\Vert \varvec{\delta }_h\Vert _{{\mathbf {L}}^2(\varOmega )}} \end{aligned}$$

124 $$\begin{aligned}&\qquad + \mathop {\mathop {\sup }\limits _{\varvec{\eta }_h \in \mathbf {\Theta }_h}}\limits _{v_h \in W_h} \frac{\langle \mathbf {{div}}\left( \mathcal {I}_{\underline{\mathbf {M}}}\underline{\mathbf {m}}- \underline{\mathbf {m}}_h\right) , \varvec{\eta }_h\rangle + \int _{\varOmega } \left( \mathcal {I}_{\mathbf {\Theta }}\varvec{\gamma }- \varvec{\gamma }_h \right) \cdot (\nabla v_h - \varvec{\eta }_h)\, dx}{\Vert \varvec{\eta }_h, v_h\Vert _{\mathbf {\Theta }\times W,t}} \end{aligned}$$

The first term, (122), was treated in [40], and is bounded by

125 $$\begin{aligned} (122) \le c \left( \sum _{T\in \mathcal {T}_h} h^{2m} \left( \Vert \varvec{\theta }\Vert ^2_{{\mathbf {H}}^{m+1}(T)}+\Vert \underline{\mathbf {m}}\Vert ^2_{{\underline{\mathbf {H}}}^{m}(T)}\right) \right) ^{1/2} \end{aligned}$$

For the second term (123), we used the commuting diagram property of the interpolation operators $\mathcal {I}_{W}$ and $\mathcal {I}_{\mathbf {\Theta }}$ (111) and the linearity of $\mathcal {I}_{\mathbf {\Theta }}$,

126 $$\begin{aligned} (123)= & {} \sup _{\varvec{\delta }_h\in \mathbf {\Gamma }_h} \frac{\int _\varOmega \left( \mathcal {I}_{\mathbf {\Theta }}\nabla w-\nabla w_h - \mathcal {I}_{\mathbf {\Theta }}\varvec{\theta }+ \varvec{\theta }_h + \frac{t^2}{\mu }(\mathcal {I}_{\mathbf {\Theta }}\varvec{\gamma }-\varvec{\gamma }_h)\right) \cdot \varvec{\delta }_h\,dx }{t\Vert \varvec{\delta }_h\Vert _{{\mathbf {L}}^2(\varOmega )}}\qquad \qquad \end{aligned}$$

127 $$\begin{aligned}= & {} \sup _{\varvec{\delta }_h\in \mathbf {\Gamma }_h} \frac{\int _\varOmega \left( \mathcal {I}_{\mathbf {\Theta }}\left( \nabla w-\varvec{\theta }+ \frac{t^2}{\mu } \varvec{\gamma }\right) -\left( \nabla w_h - \varvec{\theta }_h - \frac{t^2}{\mu }\varvec{\gamma }_h\right) \right) \cdot \varvec{\delta }_h\,dx }{t\Vert \varvec{\delta }_h\Vert _{{\mathbf {L}}^2(\varOmega )}}. \end{aligned}$$

The discrete solution $\varvec{\gamma }_h$ satisfies $\varvec{\gamma }_h = \mu t^{-2} (\varvec{\theta }_h - \nabla w_h)$ (see 102). As the solution $\varvec{\gamma }$ satisfies $\varvec{\gamma }= \mu t^{-2} (\varvec{\theta }- \nabla w)$, we obtain

128 $$\begin{aligned} (123) = 0. \end{aligned}$$

We proceed to estimating the last term (124). Since $(\underline{\mathbf {m}}_h, \varvec{\gamma }_h)$ and $(\underline{\mathbf {m}}, \varvec{\gamma })$ are solutions to the discrete and infinite-dimensional variational equations, and since $(\varvec{\eta }_h, w_h) \in \mathbf {\Theta }_h \times W_h \subset \mathbf {\Theta }\times W$, we have

129 $$\begin{aligned} \langle \mathbf {{div}}(\underline{\mathbf {m}}- \underline{\mathbf {m}}_h), \varvec{\eta }_h\rangle + \int _{\varOmega } (\varvec{\gamma }-\varvec{\gamma }_h)\cdot (\nabla v_h - \varvec{\eta }_h)\, dx = 0. \end{aligned}$$

The first term above and the divergence of similar differences of $\underline{\mathbf {m}}$ and discrete tensors in $\underline{\mathbf {M}}_h$ is well-defined in the sense

130 $$\begin{aligned} \langle \mathbf {{div}}(\underline{\mathbf {m}}- \underline{\mathbf {m}}_h), \varvec{\eta }_h\rangle = \langle \mathbf {{div}}\underline{\mathbf {m}}, \varvec{\eta }_h\rangle _{\mathbf {\Theta }^*\times \mathbf {\Theta }} - \langle \mathbf {{div}}\underline{\mathbf {m}}_h, \varvec{\eta }_h\rangle _{\mathbf {\Theta }_h^*\times \mathbf {\Theta }_h}. \end{aligned}$$

We may rewrite

131 $$\begin{aligned} (124) = \mathop {\mathop {\sup }\limits _{\varvec{\eta }_h \in \mathbf {\Theta }_h}}\limits _{v_h \in W_h} \frac{\langle \mathbf {{div}}(\mathcal {I}_{\underline{\mathbf {M}}}\underline{\mathbf {m}}- \underline{\mathbf {m}}), \varvec{\eta }_h\rangle + \int _{\varOmega } (\mathcal {I}_{\mathbf {\Theta }}\varvec{\gamma }- \varvec{\gamma })\cdot (\nabla v_h - \varvec{\eta }_h)\, dx}{\Vert \varvec{\eta }_h, v_h\Vert _{\mathbf {\Theta }\times W,t}} \end{aligned}$$

In [40] we have shown that

132 $$\begin{aligned} \langle \mathbf {{div}}(\mathcal {I}_{\underline{\mathbf {M}}}\underline{\mathbf {m}}- \underline{\mathbf {m}}), \varvec{\eta }_h\rangle \le c \Vert \mathcal {I}_{\underline{\mathbf {M}}}\underline{\mathbf {m}}- \underline{\mathbf {m}}\Vert _{\underline{\mathbf {M}}_h} \Vert \varvec{\eta }_h\Vert _{{\mathbf {H}}({\text {curl}})}. \end{aligned}$$

Thus, we deduce

133 $$\begin{aligned}&(124) \end{aligned}$$

134 $$\begin{aligned}&\le c \mathop {\mathop {\sup }\limits _{\varvec{\eta }_h \in \mathbf {\Theta }_h}}\limits _{v_h \in W_h} \frac{\Vert \mathcal {I}_{\underline{\mathbf {M}}}\underline{\mathbf {m}}- \underline{\mathbf {m}}\Vert _{\underline{\mathbf {M}}_h} \Vert \varvec{\eta }_h\Vert _{{\mathbf {H}}({\text {curl}})} +\Vert \mathcal {I}_{\mathbf {\Theta }}\varvec{\gamma }- \varvec{\gamma }\Vert _{{\mathbf {L}}^2} \Vert \nabla v_h - \varvec{\eta }_h\Vert _{{\mathbf {L}}^2}}{\Vert \varvec{\eta }_h, v_h\Vert _{\mathbf {\Theta }\times W,t}} \end{aligned}$$

135 $$\begin{aligned}&\le c \left( \Vert \mathcal {I}_{\underline{\mathbf {M}}}\underline{\mathbf {m}}- \underline{\mathbf {m}}\Vert _{\underline{\mathbf {M}}_h} + t\Vert \mathcal {I}_{\mathbf {\Theta }}\varvec{\gamma }- \varvec{\gamma }\Vert _{{\mathbf {L}}^2(\varOmega )}\right) \end{aligned}$$

136 $$\begin{aligned}&\le c \left( \sum _{T\in \mathcal {T}_h} h^{2m} \Vert \underline{\mathbf {m}}\Vert ^2_{{\underline{\mathbf {H}}}^{m}(T)} + t^2\Vert \varvec{\gamma }\Vert _{{\mathbf {H}}^m(T)}^2 \right) ^{1/2}. \end{aligned}$$

Consequently, we have arrived at the desired result. $\square$

### Hybridization

To avoid the implementation of normal–normal continuous finite elements and an indefinite system matrix, a hybridization technique in the spirit of [13, Chapter 7.1] was mentioned in [38] and analyzed in [43]. Here, the normal–normal continuity of the tensor of bending moments is broken and imposed by Lagrangian multipliers defined on element edges. The Lagrangian multipliers resemble the normal component of the rotation $\gamma _n$. As the Lagrangian multipliers are chosen of the same polynomial order as the normal–normal component of the bending moment, the discrete systems are equivalent. However, now the bending moment $\underline{\mathbf {m}}$ is completely local and can be eliminated element-wise (static condensation). The remaining system contains only displacement-based unknowns. It is symmetric positive definite, which makes it easier to be solved by sparse direct solver or standard iterative solvers.

## Numerical example

### Clamped square plate

The first example is taken from [23], where the solution is known analytically. We consider a clamped square plate $\varOmega = (0,1)^2$, i.e. at the boundary deflection $w = 0$ and rotation $\varvec{\theta }= 0$ vanish. The plate thickness varies from $t=0.1$ to $t= 10^{-5}$. Young’s modulus and Poisson ratio are chosen as $E = 12$ and $\nu = 0$. The shear correction factor is set to $k_s = 5/6$. The vertical component of the volume load is chosen as

137 $$\begin{aligned}&f_z(x, y) \nonumber \\&\quad =\frac{E}{1-\nu ^2} \Big ( y(y-1)(5x^2-5x+1)\big (2y^2(y-1)^2+x(x-1)(5y^2-5y+1)\big ) \nonumber \\&\qquad +\, x(x-1)(5y^2-5y+1)\big (2x^2(x-1)^2+y(y-1)(5x^2-5x+1)\big ) \Big ).\quad \end{aligned}$$

The solution $(\varvec{\theta },w)$ is given by

138 $$\begin{aligned} \theta _1(x,y)= & {} y^3(y-1)^3x^2(x-1)^2(2x-1), \end{aligned}$$

139 $$\begin{aligned} \theta _2(x,y)= & {} x^3(x-1)^3y^2(y-1)^2(2y-1), \end{aligned}$$

140 $$\begin{aligned} w(x,y)= & {} \frac{1}{3} x^3(x-1)^3y^3(y-1)^3 \nonumber \\&-\,\frac{2t^2}{5(1-\nu )} \big (y^3(y-1)^3x(x-1)(5x^2-5x+1) \nonumber \\&+\,x^3(x-1)^3y(y-1)(5y^2-5y+1) \big ). \end{aligned}$$

Two discretization methods are compared: the MITC7 element [16] and the TDNNS element for $k=1$ and $k=2$. In case of the MITC7 element and the TDNNS element with $k=1$, the deflections are discretized by polynomials of order two. For the higher-order TDNNS element, the deflections are of order three.

**Fig. 1:**
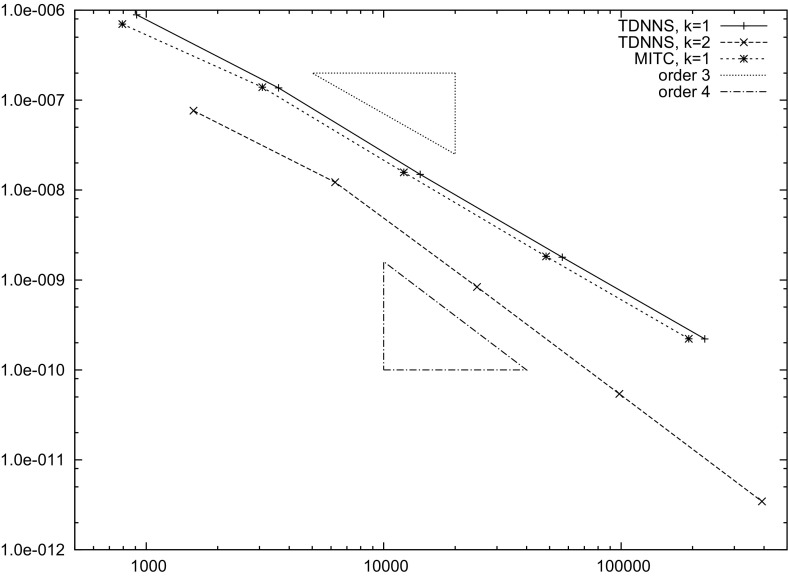
Convergence of $\Vert w-w_h\Vert _{L^2}$ over the number of degrees of freedom for the MITC7 element as well as the TDNNS element for $k=1$ and $k=2$, thickness $t=10^{-3}$

**Fig. 2:**
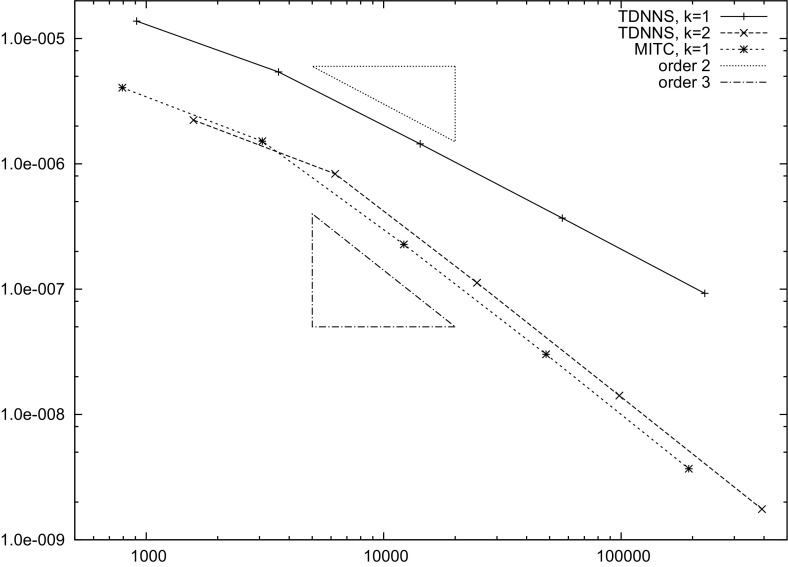
Convergence of $\Vert \varvec{\theta }-\varvec{\theta }_h\Vert _{{\mathbf {L}}^2}$ over the number of degrees of freedom for the MITC7 element as well as the TDNNS element for $k=1$ and $k=2$, thickness $t=10^{-3}$

First, we compare the different methods for a thickness of $t=10^{-3}$. In Fig. 1, the convergence of $\Vert w-w_h\Vert _{L^2(\varOmega )}$ is shown, Fig. 2 displays the convergence of $\Vert \varvec{\theta }-\varvec{\theta }_h\Vert _{{\mathbf {L}}^2(\varOmega )}$. It shows that for the deflection *w*, both the MITC7 element and the lowest-order TDNNS element with $k=1$ show convergence order three, while the TDNNS element with $k=2$ converges, as expected, at order four. However, for the rotations $\theta$, the MITC7 element and the TDNNS element with $k=2$ converge at the same rate of order three, while the lowest-order TDNNS element shows a convergence rate of order two. Thus, from the point of view of convergence, the MITC7 element lies in between the TDNNS elements with $k=1$ and $k=2$.

**Fig. 3:**
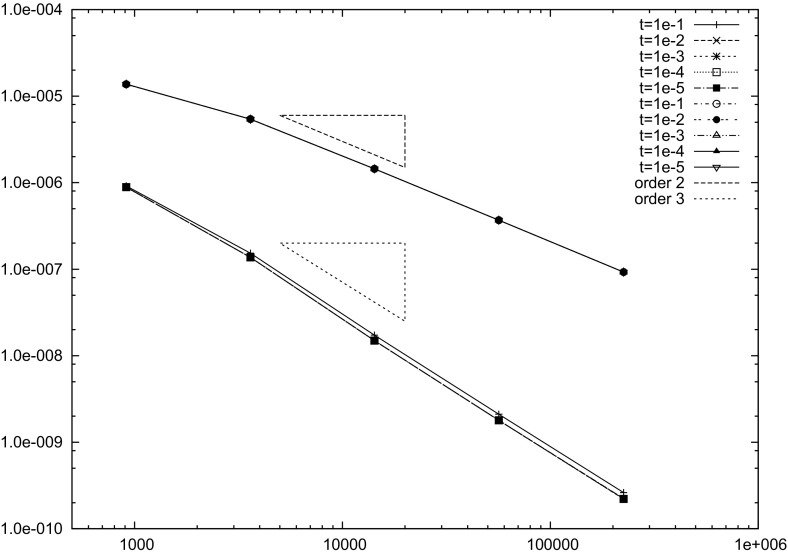
Convergence of $\Vert w-w_h\Vert _{L^2}$ and $\Vert \varvec{\theta }-\varvec{\theta }_h\Vert _{{\mathbf {L}}^2}$ over the number of degrees of freedom for the TDNNS element for $k=1$, thickness *t* varying from $t=0.1$ to $t=10^{-5}$

Next, we plot the convergence of the lowest order TDNNS method for different thicknesses. Figure 3 shows the convergence of the method for thicknesses varying between $t=0.1$ and $t=10^{-5}$. The error curves are very close, as the method does not suffer from the degrading thickness.

**Fig. 4:**
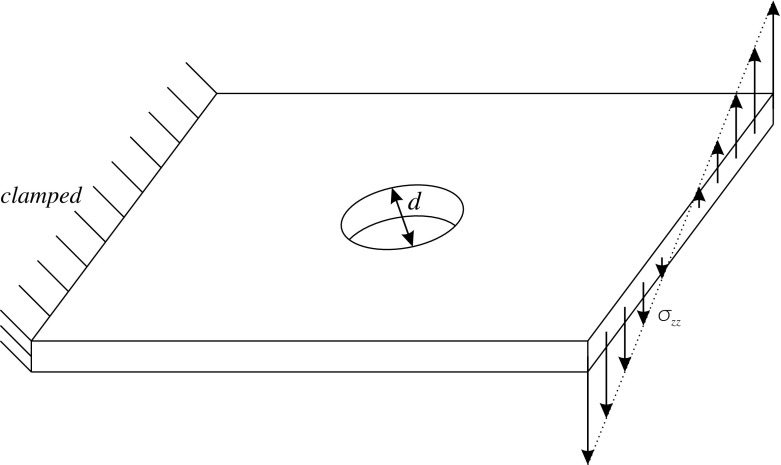
Sketch of the setup for the plate with hole

**Fig. 5:**
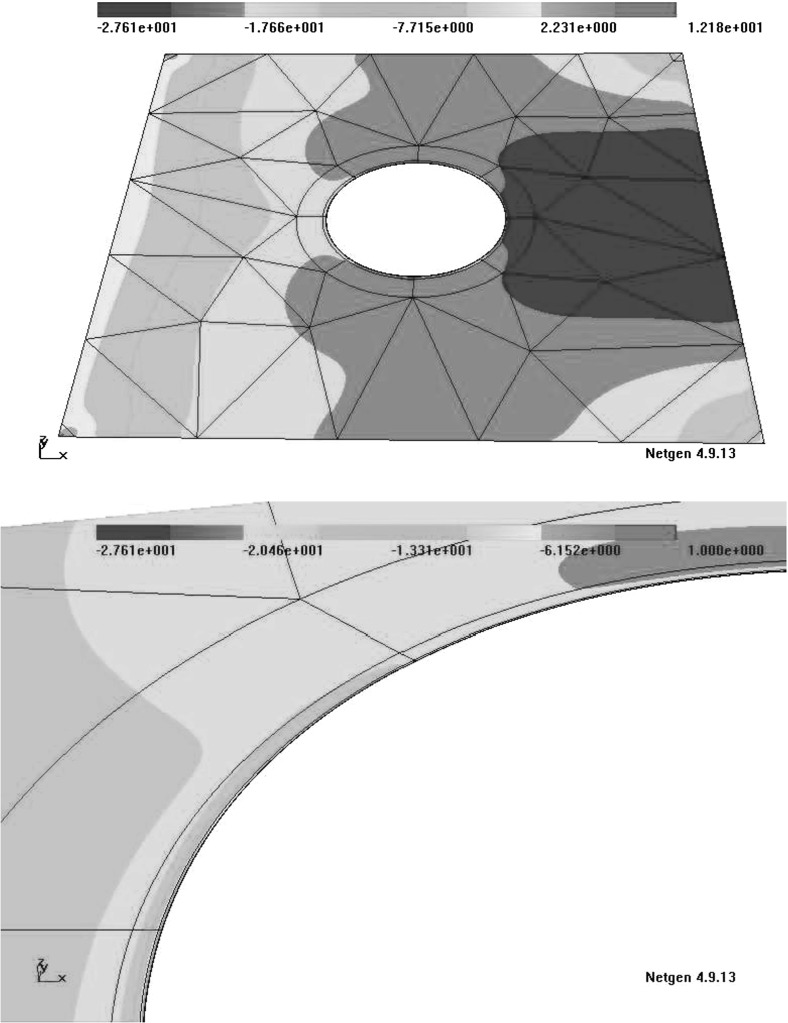
Bending moment $\underline{\mathbf {m}}_{xy}$, using TDNNS elements with $k=4$. A different *scale* is used in the zoom to the interior hole to make visible the steep gradient of the bending moment

**Fig. 6:**
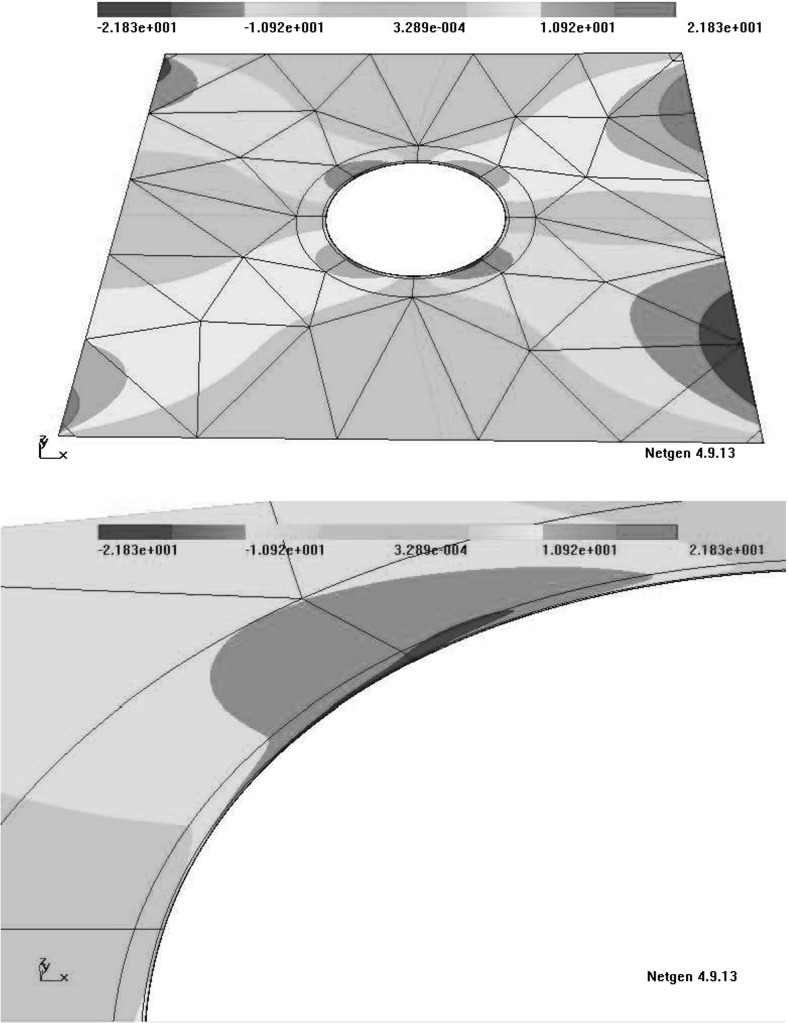
Bending moment $\underline{\mathbf {m}}_{yy}$, using TDNNS elements with $k=4$

### Square plate with hole

In the second example, we consider a square plate of dimensions $100 \times 100$ mm, in which a circular hole of diameter $d = 30$ mm is cut. The Young’s modulus $E = 2.1\times 10^5$ N/mm$^2$ and Poisson ratio $\nu = 0.3$ are chosen as those of steel. The shear correction factor is set to $k_s = 5/6$. The plate is clamped at the left hand side ($x=0$), and a surface traction $\sigma _{zz} = 0.1(y-50)$ N/mm$^2$ acts on the right hand side. All other boundaries are free. See Fig. 4 for a sketch of the setup.

An initial mesh consisting of 56 elements of mesh size approximately $h = 30$ mm is used. A two-level geometric refinement towards the corners and the free boundary at the center hole is applied to catch singularities, leading to a total number of 95 elements. The TDNNS method with $k=4$ is applied, which leads to 2593 coupling degrees of freedom.

The bending moments $\underline{\mathbf {m}}_{xy}$ and $\underline{\mathbf {m}}_{yy}$ are depicted in Figs. 5 and 6, respectively. Note that in Fig. 5, different scales are used for the original plate and the zoom to the interior hole, such that the steep gradient of the bending moment becomes visible.
